# Supplement Abstracts

**DOI:** 10.1002/jfa2.70099

**Published:** 2026-02-02

## 1 Evaluation of Patient Outcomes Following Implementation of an Alternative, Community‐Based Model of Care for Complex High Risk Foot Patients During the Covid‐19 Pandemic

Liz Perry ^1,^*, Shan Bergin^2^, Eldho Paul^3^, Naomi Rowlings^1^, Carly Bertram^1^, Nalini Natesan^4^, Julia Gilmartin‐Thomas^2,5^

^1^ *Podiatry, Alfred Health, Melbourne, Victoria, Australia*, ^2^ *School of Allied Health, Human Services & Sport, La Trobe University, Bundoora, Victoria, Australia*, ^3^ *School of Public Health and Preventive Medicine, Monash University, Melbourne, Victoria, Australia*, ^4^ *Clinical Governance, Alfred Health, Melbourne, Victoria, Australia*, ^5^ *Allied Health Department, Alfred Health, Melbourne, Victoria, Australia*

*L.Perry@alfred.org.au

**Background:** Extreme pressure on acute healthcare settings during the Covid‐19 pandemic resulted in the rapid implementation of alternative health service models. In 2020, complex high risk foot patients attending hospital based, podiatry outpatient services at Alfred Health, were transferred to community‐based services to alleviate acute service pressure and mitigate risk of infection.

**Aim:** This study aimed to evaluate the impact of this transition on outcomes including emergency presentations, wound numbers, podiatry service use, amputation rates, numbers lost to follow up, and deaths.

**Methods:** A retrospective audit of electronic medical records was conducted for all patients transferred from acute to community‐based care between April 2020 and July 2020. Using the Australian Clinical Triage Guide for People with Diabetes Related Foot Disease, patients categorised as ‘highly serious’, ‘serious’ or ‘stable’, were transferred to community based podiatry services. Data were collected for a 6 month period pre and post transfer. Demographic and clinical data including age, gender, diabetes type, vascular and renal status, foot ulcer and amputation history was collected at which time a comparative analysis was undertaken.

**Results:** In total, 57 patients were transferred to community based podiatry services during the study period. Of these, 22 were ‘highly serious’, 28 ‘serious’, and 7 ‘stable’. Of the 57 patients, 93% had diabetes, 11% end stage renal failure, 60% peripheral arterial disease, 9% acute Charcot foot and 47% had foot ulcers. There was no significant difference in service use (podiatry visits), escalation of care (to emergency and/or hospital medical units), amputation and death pre and post transfer, across the total cohort. However when comparing outcomes for the ‘highly serious’ group, to outcomes for the ‘serious’ and ‘stable’ groups, there were significantly higher rates of both service use and escalation of care (podiatry visits, emergency visits for a foot complication, and escalation to medical units).

**Conclusions:** This study highlights the importance of hospital‐based care for podiatric patients classified as ‘highly serious’, compared to patients classified as ‘serious’ or ‘stable’. This study identified that patients classified as serious or stable could safely access care with community‐based podiatry services without significantly impacting patient outcomes.

## 2 Osteoarthritis Chronic Care Program (OACCP): The Importance of Podiatry Involvement in the Multidisciplinary Care of Patients With Knee and Hip Osteoarthritis

Lily Nguyen ^1^, Vanessa Nube^1^, Priya Gnanakumaran^2^, Richard Holland^3^, Ananthila Anandacoomarasamy^3^

^1^ *Department of Podiatry, Sydney Local Health District, Camperdown, New South Wales, Australia*, ^2^ *Concord Osteoarthritis Chronic Care Program, Sydney Local Health District, Concord, New South Wales, Australia*, ^3^ *Department of Rheumatology, Sydney Local Health District, Camperdown, New South Wales, Australia*

**ABSTRACT**

Osteoarthritis (OA) affects one in four Australians over 65, and poses a significant burden to individuals, health systems and the economy. In NSW, Joint Replacement Surgeries (JRS) grew by 50% between 2005 and 2015, with 70% of patients undergoing JRS having not accessed conservative care, despite evidence‐based guideline recommendations. The Osteoarthritis Chronic Care Program (OACCP) funded by NSW Government, is an evidence‐based, multidisciplinary team model of care involving physiotherapy and exercise, weight loss, pain management and psychological care. The OACCP aims to improve function and quality of life for patients awaiting JRS or opting for conservative care, potentially reducing surgeries by 10%. A 2023 cross‐sectional study of 26,003 participants with symptomatic knee or hip OA found 12% reported foot pain. Despite this, podiatry involvement throughout OACCP clinics is limited to 1 out of 26 in NSW. Within this clinic, 50% of patients access podiatry services. The aim of these three case reports is to highlight the role of Podiatry in the OACCP. A 67‐year‐old woman with knee OA, awaiting JRS, was unable to engage in exercise due to heel pain. Orthotic modifications to address her out‐of‐phase pronation alongside exercises targeting gastrocnemius strength, resolved her Achilles tendinopathy‐related heel pain, enabling her to participate successfully in her knee‐targeted home exercise program (HEP). A 51‐year‐old man with knee OA and chronic bilateral ankle pain hindering daily activity was provided footwear advice to improve his out‐of‐phase pronation and a HEP. This led to substantial improvement in ankle pain, enabling his return to independent function and measurable improvement in his quality of life. A 74‐year‐old female with moderate to severe knee OA awaiting JRS, was recommended to replace her unsupportive leisure shoes with a supportive and stable sports shoe and prescribed a HEP. This resulted in reduced knee‐pain, increased mobility, and an improved quality of life, leading her to cancel her surgery in favour of conservative management. These cases highlight the Podiatrist's role in improving outcomes for patients living with lower‐limb OA. Further data is needed to advocate for improved access to podiatry consultation within multidisciplinary teams such as OACCP.

## 3 Clinical Application of Elastic Corrective Nail Surface Wire in the Non‐Surgical Treatment of Ingrown Toenails

Riliang Cao^1^, Davy Wei Dai^2^, Qionglin Liu^1^, Yan Cheng^1^, Fangmin Li

^1^ *Department of Rehabilitation Therapy, School of Health, Guangzhou Vocational*, ^2^ *Technical University of Science and Technology*

**ABSTRACT**

**Objective:** This study aims to investigate the clinical efficacy of elastic corrective nail surface wire in treating ingrown toenails (onychocryptosis).

**Methods:** From June 2022 to June 2023, 20 patients (30 toenails; 12 males and 8 females) with ingrown toenails, aged 11–73 years (mean age: 29.17 ± 17.05 years), were treated with elastic corrective nail surface wires. The treatment outcomes were followed up for 1 year. Statistical analyses were conducted on the pre‐ and post‐treatment pain VAS scores (maximum score: 10), post‐treatment appearance satisfaction VAS scores (maximum score: 10), the recurrence rate of curved nail deformities after 1 year, and the occurrence of complications (e.g., onychomadesis, subungual haematoma and wire dislodgment).

**Results:** The mean pre‐treatment VAS score was 6.37 ± 2.51. Immediate pain relief was observed after treatment, with a post‐treatment pain VAS score of 2.97 ± 2.09, showing a statistically significant difference (*p* < 0.05). At the 1‐year follow‐up, 18 cases were cured (cure rate: 90%), and 2 cases experienced recurrence (recurrence rate: 10%). No cases of nail detachment, subungual haematoma, or wire dislodgment were reported. Two patients (three toenails) experienced a recurrence of curved nail deformities but without pain. The post‐treatment appearance satisfaction VAS score was 9.23 ± 1.14 for all cases.

**Conclusion:** Compared to the traditional invasive surgical treatment of ingrown toenails, elastic corrective nail surface wire offers a non‐invasive alternative with significant long‐term clinical efficacy. It is recommended for clinical promotion and application.

## 4 Early Diagnosis of Charcot Neuro‐Osteoarthropathy Using MRI and Its Effect on Patient Outcomes: A 7‐Year Retrospective Audit

Deborah Schoen ^1,^*, Laksh Lukkhoo^1^, Sharlene Vu^1^, Joanna Scheepers^2^

^1^ *Podiatric Medicine and Surgery Discipline, The University of Western Australia, Crawley, Western Australia, Australia*, ^2^ *St John of God Midland Public and Private Hospital, Perth, Western Australia, Australia*

*deborah.schoen@uwa.edu.au

**Background:** Charcot neuro‐osteoarthropathy is a debilitating condition characterised by inflammatory processes in individuals with peripheral neuropathy, predominantly affecting the foot and ankle, particularly in patients with diabetes. There remains a paucity of research comparing the diagnostic and therapeutic outcomes between Magnetic Resonance Imaging (MRI) and X‐ray modalities for Charcot neuro‐osteoarthropathy. This retrospective study investigates the use of offloading devices, duration of offloading and final footwear outcomes dependent on imaging at diagnosis.

**Methods:** This retrospective audit was conducted over 5 years. Medical records from a secondary hospital high‐risk foot clinic in Perth, Western Australia, were systematically reviewed. Data collected included baseline medical history, location of Charcot neuro‐osteoarthropathy, Eichenholtz stage or Chantelau and Grutznel grade at diagnosis, type, and duration of offloading, and final footwear outcomes.

**Result:** Twenty‐eight patients met the inclusion criteria. All had diabetes (18% type 2% and 53% type 1) and peripheral neuropathy. All patients received either an MRI (43%) or X‐ray (57%) to confirm the diagnosis of active Charcot neuro‐osteoarthropathy. Five (17.9%) patients who were diagnosed on MRI had grade 0 Charcot neuro‐osteoarthropathy whilst, 23 (82.1%) patients who were diagnosed on X‐ray had stage 1 Charcot neuro‐osteoarthropathy. No statistical significance was found between the type of offloading used, duration of offloading, resolution of Charcot neuro‐osteoarthropathy, footwear outcomes and transtibial amputation outcomes across those diagnosed with MRI or X‐ray.

**Conclusion:** No statistical significance in patient outcomes was found between those diagnosed with grade 0 on MRI and those diagnosed with stage 1 on X‐ray.

## 5 Sole Stories: A Qualitative Study on Yarning About Diabetic Foot Education and Plantar Pressure Mapping With Aboriginal People

Deborah Schoen ^1,^*, Glenys Collard^1^, Steve Gillinghan^1^, Amber Saxton^1^, Lily Smith^1^, Samuel Steel^1^, Beatriz Alija‐Martinez^1^, James Charles^2^

^1^ *Podiatric Medicine and Surgery Discipline, The University of Western Australia, Crawley, Western Australia, Australia*, ^2^ *First Peoples Health Unit, Griffith University, Gold Coast, Queensland, Australia*

*deborah.schoen@uwa.edu.au

**Background:** This study aims to explore the experiences of Aboriginal people with diabetic foot disease and their families in receiving podiatry foot care. It also seeks to identify the medical jargon encountered by Aboriginal individuals with diabetic foot disease and their families. Additionally, the study aims to investigate Aboriginal peoples' perspectives on the value of foot pressure maps in diabetic foot disease education. Finally, the study aims to co‐design strategies for effectively communicating foot pressure maps, potentially using metaphors or narratives.

**Methods:** This study is a collaborative effort, using co‐design with Aboriginal people on Noongar Wadjuk Boodja in Perth, Western Australia, under the guidance of an Aboriginal Chief Investigator and in collaboration with an Aboriginal Advisory Group. Qualitative methods involving one‐on‐one research yarns to discuss diabetic foot disease education and plantar pressure maps will be used to gather data from Aboriginal individuals with diabetic foot disease and their families. Data collected will be analysed to identify common experiences, encounters with medical jargon, and perspectives on plantar pressure maps for offloading education. The participants and the Aboriginal Advisory Group will review all data. The Noongar Nation will own the data as per the Data Sovereignty principles developed by the Lowitja Institute.

**Results:** Since July 2022, we have engaged with eight Aboriginal academics, two Aboriginal Healthcare Workers and seven Aboriginal health organisations. Initial community engagement focused on reviewing the protocol and recruiting members for our Aboriginal Advisory Group. Researchers have attended meetings with Aboriginal Community groups, spreading the word about our project, and recruited seven members to our Advisory Group, including Elders. Careful documentation of meetings has modified the research protocol to enhance cultural safety and ensure ongoing co‐design.

**Conclusions:** This study, which addresses the critical health issue of higher rates of diabetic foot disease and amputation in Aboriginal Australian people, has the potential to make a significant impact. We are combining the cultural practice of earning with the visual representation of plantar pressure maps for offloading, a strategy that respects Aboriginal peoples' way of knowing, being, and doing.

## 6 Total Contact Casting Duration for the Clinical Remission of Active Charcot Neuro‐Osteoarthropathy: A Retrospective Study

Deborah Schoen ^1,^*, Kaitlyn Buffon^1^, Sabiha Khan^1^, David King^1^, Matthew Pranoto^1^, Mendel Baba^2^

^1^ *Podiatric Medicine and Surgery Discipline, The University of Western Australia, Nedlands, Western Australia, Australia*, ^2^ *Podiatry Department, Sir Charles Gairdner Osborne Park Health Care Group, Nedlands, Western Australia, Australia*

*deborah.schoen@uwa.edu.au

**Background:** Charcot neuro‐osteoarthropathy is a severe foot and ankle joint disease that can result in deformities, ulcers, and lower extremity amputation. Total contact casting is the gold standard treatment for offloading and immobilising the foot; however, there is no universally agreed upon total contact casting treatment duration, leading to inconsistencies in published data. This study aims to identify the duration of TCC treatment for clinical remission of active Charcot neuro‐osteoarthropathy.

**Methods:** This retrospective audit reviewed paper records of all patients with active Charcot neuro‐osteoarthropathy, without ulceration, treated using total contact casting over a 5 year period between 1 January 2019 and 31 December 2023 at a tertiary hospital high risk foot service in Western Australia. Descriptive statistics were used including medians and interquartile ranges (IQR) for continuous data and proportions for categorical variables. Non‐parametric tests were employed to explore group differences and total contact casting durations.

**Results:** A total of 12 records met the inclusion/exclusion criteria. The median age of patients was 59.5 years (IQR, 51.25–70.25), with 58.3% being male. All patients had peripheral neuropathy, and 91.7% of them had diabetes. Most participants (83.3%) were at stage 1 Charcot neuro‐osteoarthropathy, using the Eichenholtz classification system. The median total contact casting duration for achieving clinical remission of active Charcot neuro‐osteoarthropathy was 88.5 days (IQR, 65–173.3). No significant associations were found between variables and total contact casting durations.

**Conclusions:** The median total contact casting treatment duration for achieving clinical remission of active Charcot neuro‐osteoarthropathy was 88.5 days, which is shorter than the durations reported in previous studies. These findings contribute to existing data and can aid clinicians in managing and setting realistic patient expectations regarding the timeline for total contact casting duration ultimately leading to better patient care and outcomes.

## 7 Measuring High‐Risk Foot Patient Experiences and Satisfaction Through Their Journey in the Australian Tertiary Hospital Clinic: A Mixed Method Study

Deborah Schoen ^1,^*, Gareth Cooper^1^, Anthony Gielens^1^, Alinda Lee^1^, Damyn Packwood‐Tuhakaraina^1^, Cara Westphal^2^, Kirsten Nielsen^2^

^1^ *Podiatric Medicine and Surgery Discipline, The University of Western Australia, Crawley, Western Australia, Australia*, ^2^ *Royal Perth Bentley Group Podiatry Department, Royal Perth Hospital, Perth, Western Australia, Australia*

*deborah.schoen@uwa.edu.au

**Background:** To assess patient experiences and perspectives with care received at an Australian tertiary hospital high‐risk foot clinic and the care's impact on the patients' reported quality of life (QoL).

**Methods:** A mixed methods cross‐sectional study recruited patients attending the tertiary hospital high‐risk foot clinic over 5‐months and assessed the patients using the Wound‐QoL‐17 tool, a patient satisfaction survey, and the Australian Diabetic Foot Minimum Dataset. Participant Wound‐Qol‐17 responses were individually scored, and the global wound score is an average of all item answers. The participants' perceptions of the staff, quality of care, and overall perception of the clinic were thematically analysed to extract core themes. Pearson correlation analysis was used to analyse the correlation between global wound score, staff satisfaction, clinic rating, and patient understanding. Statistical significance was *p* < 0.05.

**Results:** Eighty‐five patients attending the clinic participated in the study, with an average age of 63.9 (± 13.9) and 83.5% male. The total global wound‐QoL‐17 score had a median of 2.3 with a 95% CI of [2.3, 2.7]. The median values of the wound‐QoL‐17 subscales were 1.8 with 95% CI [1.8, 2.1] for ‘Body’, 2.8 with 95% CI [2.6, 3.1] for ‘Psyche’, and 2.2 with 95% CI of [2.4, 2.9] for ‘Everyday Life’. The patient satisfaction reported a high satisfaction level with the clinic and staff, with common themes of improvement relating to accessibility (24%). Correlations between global wound score and patient satisfaction themes of staff satisfaction, clinic rating, and patient understanding were statistically insignificant when comparing the wound‐Qol‐17 to any aspect of the patient satisfaction survey. A moderate positive correlation between the Clinic rating and patients' satisfaction with the clinic staff was found, and a high positive correlation between a patients' involvement in their treatment journey and patients' satisfaction with the clinic staff.

**Conclusions:** The observed relationship between the levels of patient satisfaction and their quality of life was too slight to infer a definitive conclusion, and would benefit from further research studies involving larger populations would be beneficial. Overall, the high‐risk foot clinic was highly rated, and patient satisfaction and clinic rating increased with patients' understanding their health journey.

## 8 Podiatry Resources in the Top End—A Total Croc! Tales From Darwin's Only Community Funded Podiatrist

Anna Stybowski

*Danila Dilba Biluru Binnilutlum Health Services, Garramilla – Larrakia Country (Darwin), Northern Territory, Australia*

Anna.stybowski@ddhs.org.au

The Northern Territory shamefully boasts the highest lower limb amputation rates in all of Australia. The reasons for this are complex, and stem from the brutal intergenerational effects of British colonisation and dispossession of Australia's First Nations people. Despite the complexity of poorly managed diabetes and its sequelae—as podiatrists, we know very well that there is hope for lower limb preservation and maintenance of good foot health. But we, and the communities we serve, need the right resources. In the Top End, health professionals and community face multiple complex barriers to accessing these resources.

Danila Dilba Health Service is an Aboriginal Community Controlled Health Organisation based in Garramilla (Darwin). Alarmingly, despite the high rate of diabetic foot complications in the region, Danila Dilba has only recently added Podiatry to its suite of health and wellbeing services. The podiatry service has been operating for just under 4 years, at 4 days per week, and already boasts the highest demand, receiving more referrals than any other service at Danila Dilba. Danila Dilba's Podiatry service is also the only community‐based Podiatry service in Darwin—a reflection of the historic under resourcing in the Top End.

I would like to share the story of how Podiatry was established at Danila Dilba Health Service and the impacts the service has had on Danila Dilba and the local community, including the impacts and experiences of working alongside an Aboriginal Allied Health Assistant. I would also like to share my own reflections and experiences as a Podiatrist with a white privileged background, working in an ACCHO on Larrakia Country—what I have learnt about local culture, myself, my profession and the privilege of good foot health.

This work is part of the South Australian Health and Medical Research Insititute's Aboriginal and Torres Strait Islander Diabetic Foot Complications Program.

## 9 Amputation Resources for Aboriginal South Australians

Courtney Hammond*, Matthew Kemsley, Saraid Martin, Kim Morey

*South Australian Health and Medical Research Institute (SAHMRI), Adelaide, South Australia, Australia*

*Courtney.hammond@sahmri.com

**Background:** The rate of lower limb amputation in South Australia during 2011–2018 for Aboriginal people was 3.4 times greater than the non‐Aboriginal population rate and accounted for 4.9% of all amputations. Health care teams acknowledge that a loss of limb can be a life changing event with inequities in access to services creating barriers that lead to difficulties engaging Aboriginal patients experiencing an amputation. Previous work from SAHMRI found that the amputation journey for Aboriginal patients in South Australia is complex and variable, yet stories of strength and determination was evident. Further, this report detailed 40 recommendations for targeted health system improvements across each phase of the amputation journey for Aboriginal South Australians. The aim of this project is to implement the report recommendation regarding development of culturally appropriate amputation resources, as to date there are no suitable resources available in South Australia.

**Methods:** A co‐design, Aboriginal lived‐experience led resource development project has commenced to create culturally informed decision‐making resources to support informed choices along a person's amputation journey. The resources will be used across South Australia, central Australia and western NSW and will be freely available to clinicians, service providers, and community. Aboriginal knowledge and experience will be privileged throughout, with expertise sought from clinicians caring for those experiencing amputation, and communication, design and production experts. Throughout the project, staff utilise reflective practice to capture progress.

**Results:** To date a lived‐experience, culturally safe workshop was convened with 10 community members attending. Community member shared their stories, and articulated priorities for where additional resources would have aided their amputation journey. Subsequently, a clinician workshop was held, with 25 multi‐disciplinary clinicians attending to confirm the evidence base aligning with the most pressing priorities identified by the lived‐experience group. A communication company has analysed the data and produced a communication and dissemination strategy. By the Conference date, film and other resources production will have been completed and able to be shared.

**Conclusions:** The amputation journey is difficult, complex, and distressing for Aboriginal people in South Australia. This project addresses one aspect of amputation care that has been shown to be deficient by delivering co‐designed, culturally informed decision‐making resources.

## 10 ‘Walk Strong, Walk Tall’—Foot Health Workforce Development

Natalie Morgan*, Courtney Hammond, Kim Morey

*South Australian Health and Medical Research Institute (SAHMRI), Adelaide, South Australia, Australia*

**Background:** The Walk Strong, Walk Tall (WSWT) program, initiated by SAHMRI, addresses the limited access to appropriate foot health care for diabetes‐related foot complications (DRFC) across South Australia, including rural and remote areas. The percentage of podiatrists is significantly lower than the national average in rural and remote areas, and fewer undergraduate students are entering podiatry programs. Aboriginal South Australians suffer from DRFC, including amputations, at disproportionate rates, and the WSWT program aims to build the capacity of local clinicians, especially Aboriginal health workers, to identify and manage DRFC.

**Methods:** The program targets non‐podiatry clinicians, offering training that is adapted to the needs of individual workplaces. The training is mainly directed at Aboriginal health practitioners but is open to all clinicians involved in diabetic foot care. The training involves mixed methods to cater to diverse learning styles and workplace contexts. It is also aligned with quality improvement initiatives in clinical units. Pre‐ and post‐training surveys assess the changes in participants' knowledge and confidence regarding DRFC care. To increase the impact, the training content has been modified to meet the Australian skills framework requirements. A partnership with a registered training organisation has enabled the certification of participants in basic foot care (CHCCCS032), which is available as an elective in various healthcare qualification courses.

**Results:** Over the past 6 months, 14 training sessions were conducted, with participants reporting significant improvements in knowledge and confidence. As of 2025, Australian skills framework accredited training will be delivered, providing nationally recognised certification, with early results able to be shared at the conference.

**Conclusions:** The WSWT program demonstrates strong interest from non‐podiatry clinicians in contributing to diabetic foot care, recognising their role in multidisciplinary teams to improve healthcare outcomes, particularly for rural and remote communities. The program helps bridge healthcare gaps, complementing podiatry by supporting a collaborative, community‐focused approach to care.

## 11 Could Interprofessional Learning Experiences be Beneficial for Australian Prescribing Podiatrists and Pharmacists? A Qualitative Study

Saraid Martin ^1,^*, Kristin Graham ^1^, Helen Banwell ^1^, Jacinta Johnson ^2,3^

^1^ *Allied Health & Human Performance, University of South Australia, Adelaide, South Australia, Australia*, ^2^ *Clinical & Health Sciences, University of South Australia, Adelaide, South Australia, Australia*, ^3^ *SA Pharmacy, Statewide Clinical Support Services, SA Health, Adelaide, South Australia, Australia*

*saraid.martin@sahmri.com

**Background:** The number of podiatrists who are endorsed to prescribe is increasing. Prescribing podiatrists require mandatory continuing professional development (CPD) specific to their prescribing practice, however, CPD resources often do not meet prescribing podiatrists' needs in terms of content, relevance, accessibility, and meaningfulness. Furthermore, interprofessional collaboration, known to improve patient and organisational outcomes, requires understanding and appreciation of each other's roles. Interprofessional learning between podiatrists and pharmacists could address CPD needs and improve practice. This study aims to explore role understanding, perceptions of, and potential for, interprofessional learning between prescribing podiatrists and pharmacists.

**Methods:** Australian prescribing podiatrists and pharmacists participated in four homogenous focus groups via Zoom. Participants were asked about their opinions, feelings, experiences and knowledge relating to the roles of each discipline and interprofessional learning between the professional groups. Focus group discussions were transcribed verbatim and underwent reflexive inductive thematic analysis.

**Results:** Fifteen podiatrists and 15 pharmacists participated in the focus groups. Four themes were evident in the data: Experience drives understanding of each other; Exposure is influenced by work setting; Reflections on and frustrations with health system issues; The vision for interprofessional learning in the future. Overall, prescribing podiatrists and pharmacists were positive about the potential benefits and feasibility of interprofessional CPD to support the clinical use of medicines, build stronger relationships, and collaborative healthcare practices. Both groups acknowledged awareness of the prescribing podiatrist role and training was lacking and contributed to common frustrations with system issues. Pharmacists advocated for interprofessional learning with podiatrists at undergraduate level to align with their positive experiences with other professional groups. Suggestions for learning modes, facilitators and topics were collated.

**Conclusions:** When developing undergraduate courses, CPD and advanced training programs, podiatry and pharmacy education providers should consider the benefits of interprofessional learning and the desire of the two professional groups to learn together.

## 12 Podiatry Service Use by Colorectal Cancer Survivors With Chemotherapy Induced Peripheral Neuropathy (CIPN)

Sindhrani Dars^1,^*, Tenaw Tiruye^2^, David Roder^2^, Helen Banwell^1^, Ian Olver^3^, Kerri Beckmann^2^

^1^ *Allied Health and Human Performance, University of South Australia, Adelaide, Australia*, ^2^ *Cancer Epidemiology and Population Health Research Group and Allied Health and Human Performance, University of South Australia, Adelaide, Australia*, ^3^ *Faculty of Health and Medical Sciences, University of Adelaide, Adelaide, Australia*

*Sindhrani.dars@unisa.edu.au

**Background:** Chemotherapy Induced Peripheral Neuropathy (CIPN) impacts 68% of people receiving neurotoxic (platinum or taxane‐based) chemotherapy for breast or bowel cancer so severely it is the leading cause of reduced or ceased treatment. The implications of CIPN for lower limb health include balance deficits, falls and fall related injuries, and painful nail and skin concerns, all of which can benefit from podiatry services. The aim of this study was to explore the use of private podiatry services among Australians with colorectal cancer (CRC) and identify any predicting factors for use of podiatry services.

**Methods:** A retrospective cohort study using linked health and datasets analysed all CRC recordings in South Australia between 2011 and 2013 for patient characteristics, chemotherapy type and podiatry service use 4 years prior, and 5 years following diagnosis. Crude and adjusted Poisson regression analyses compared podiatry service use according to whether treatment included chemotherapy, and whether it was neurotoxic. Multivariable Poisson regression analysis was conducted to identify factors associated with podiatry service after CRC diagnosis.

**Results:** Of 3292 people diagnosed with CRC, 1535 (47%) received chemotherapy. The crude rate of podiatry service for chemotherapy recipients did not exceed 20% across the 5 years post‐diagnosis follow‐up. After adjusting for potential confounders, rates of podiatry service use among chemotherapy recipients remained similar to non‐recipients except in their second‐year post‐diagnosis where rates increased (incidence rate ratios [IRR] 1.22, 95% CI: 1.01–1.48). No differences in podiatry service use were observed at any timepoint between those receiving neurotoxic and non‐neurotoxic chemotherapy. Podiatry service use post‐diagnosis was positively associated with prior podiatry service use (IRR 4.08, 95% CI: 3.48–4.79), having diabetes (IRR 1.26, 95% CI: 1.01–1.57), receiving chemotherapy (IRR 1.18, 95% CI: 1.01–1.37) and older age (IRR 1.28, 95% CI: 1.07–1.53, 80+ vs. < 80 years).

**Conclusions:** Lower than expected podiatry service use by chemotherapy recipients, and lack of difference according to whether chemotherapy was neurotoxic, may indicate suboptimal follow up care for the management of CIPN in CRC patients. Improved referral pathways, analysis of tertiary centre podiatry data and inclusion of podiatrists in cancer survivorship care should be considered to ensure better management of complications from CIPN.

## 13 Custom Foot Orthoses: A Retrospective Analysis of 1000 Prescriptions From New Zealand Podiatrists

Aaron Jackson^1,2,^*, Kelly Sheerin^2^, Duncan Reid^1,2,3^, Matthew R. Carroll^1,3^

^1^ *School of Clinical Sciences, Faculty of Health and Environmental Sciences, Auckland University of Technology, Auckland, New Zealand*, ^2^ *Sports Research Institute of New Zealand, Auckland University of Technology, Auckland, New Zealand*, ^3^ *Active Living and Rehabilitation Aotearoa New Zealand, Auckland University of Technology, Auckland, New Zealand*

*aaron.jackson@aut.ac.nz

**Background:** Podiatrists frequently prescribe foot orthoses to manage a range of musculoskeletal complaints. This study aimed to understand what characteristics were included in the design of custom foot orthoses, how symmetrically these were applied, and whether there was an association between these features and the clinical diagnosis.

**Methods:** One thousand orthotic prescriptions were obtained from two commercial orthotic labs in New Zealand. Twenty‐six prescription characteristics were analysed. Descriptive data detailed the frequency of included characteristics. The symmetry feature was derived according to the characteristics of both feet and analysed considering the number of times the left foot differed from the right foot. Clinical diagnoses were grouped, and for the most common four (plantar heel pain, pes planus, posterior tibial tendon, and ankle sprain), associations between the prescription characteristics selected and diagnosis were analysed using cross tabulations and chi‐square tests.

**Results:** The most common prescription characteristics were a Polyamide 11 shell (80%), a shell thickness of 3 mm (54%), Modified Root shell style (61%) and varus cast correction (64%). Additionally, deep heel cups (36%), medial rearfoot (Kirby) skives (36%), and lateral forefoot wedges (22%) were the most prescribed modifications. Fifty‐eight percent of prescriptions were identical between the left and right sides. The most common diagnosis was plantar heel pain (11%). Plantar heel pain was associated with the characteristics of plantar fascia groove (*p* < 0.001), forefoot lateral wedge (*p* < 0.001), and heel cushion (*p* < 0.001).

**Conclusion:** Strong associations between orthotic design characteristics and diagnoses indicate consistency in prescription variables amongst New Zealand podiatrists when prescribing custom foot orthoses. Plantar heel pain and pes planus are the two clinical diagnoses for which podiatrists prescribe the most custom foot orthoses. The high degree of similarity and symmetry in the prescription of orthoses raises questions regarding the level of customisation required to exert beneficial clinical outcomes and opens a potential for future research on the topic.

## 14 The Effect of Lateral Wedging on First Metatarsophalangeal Joint Kinematics and Centre of Pressure

Aaron Jackson^1,2,^*, Kelly Sheerin^2^, SangHoon Yoon^2^, Hannah Wyatt^3^, Duncan Reid^1,2,4^, Matthew R. Carroll^1,4^

^1^ *School of Clinical Sciences, Faculty of Health and Environmental Sciences, Auckland University of Technology, Auckland, New Zealand*, ^2^ *Sports Research Institute of New Zealand, Auckland University of Technology, Auckland, New Zealand*, ^3^ *Faculty of Health, University of Canterbury, Christchurch, New Zealand*, ^4^ *Active Living and Rehabilitation Aotearoa New Zealand, Auckland University of Technology, Auckland, New Zealand*

*aaron.jackson@aut.ac.nz

**Background:** Lateral wedges are one of the most common modifications added to foot orthoses. Podiatrists frequently prescribe lateral wedges with the intention of increasing first metatarsophalangeal joint (MPJ) range of motion or altering the centre of pressure (COP). The aim of this study was to explore the effect of lateral wedging on first MPJ kinematics and COP during walking and running gait.

**Methods:** This study utilised a randomised cross‐over design in which 24 healthy participants ran and walked in 10 different insoles. The 10 insole conditions comprised differing wedge inclinations (three‐degrees vs. six‐degrees), placement (full‐length vs. forefoot) and overall contour (contoured vs. sham). First MPJ kinematics in the sagittal plane, and foot COP were examined using 3D motion analysis and an instrumented treadmill. Data were analysed across stance phase using statistical parametric mapping.

**Results:** Fifteen men and nine women, with a mean age of 37 years (SD 8.1) participated in the study. Extension of the first MPJ joint was significantly reduced by lateral wedging (*p* = 0.01), with this reduction occurring throughout the entire stance phase (0%–100%). The only instance in which inclination or placement significantly altered the outcome was during walking gait where 6‐degree wedges reduced extension significantly more than 3‐degree wedges (*p* = 0.01). Additionally, lateral wedging shifted the COP medial relative to the midline of the foot (*p* = 0.01), but this effect was only observed with six‐degree and full‐length wedges. The impact of lateral wedging was detected earlier in the stance phase for walking gait (11%–24%) compared to running gait (26%–52%).

**Conclusion:** Lateral wedges reduce extension of the first MPJ during both walking and running, regardless of wedge inclination and placement. Additionally, six‐degree and full‐length lateral wedges cause a medial shift in the COP. Clinically, if the goal is to reduce first MPJ extension, the inclination and placement of the wedge are not critical factors. However, these variables are important in relation to COP, where only the 6‐degree and full‐length wedges appear to elicit a medial shift. These findings underscore the need to design lateral wedges with consideration for the intended biomechanical outcome or the specific gait type they are meant to address.

## 15 Contemporary Multifactorial Conceptual Frameworks for Running‐Related Injury Aetiology: A Systematic Review of Qualitative Studies

Sarathadevi Thanabalu^1^, Benjamin Peterson ^1,^*, Vivienne Chuter^2^

^1^ *Department of Podiatry, School of Health, Medical and Applied Sciences, CQUniversity, Rockhampton, Queensland, Australia*, ^2^ *School of Health Sciences, Western Sydney University, Campbelltown Campus, Sydney, New South Wales, Australia*

*b.peterson@cqu.edu.au

**Background:** The aetiology of running‐related injury is widely accepted to be multifactorial, however, previous research of its risk factors has frequently focused on discrete variables. Recently, conceptual frameworks have been proposed, which aim to describe how the complex interplay between multiple factors can result in the development of a running‐related injury. The primary aim of this systematic review was to synthesise the characteristics of conceptual frameworks for the aetiology of running‐related injury which have been developed through evidence‐based approaches using qualitative research methods.

**Methods:** Electronic searches of Ovid MEDLINE, CINAHL, SPORTDiscus, EMBASE and The Cochrane library from January 2010 until 4 June 2024 were performed to identify qualitative studies which described a multifactorial conceptual framework for the development of running‐related injury. Individual frameworks were appraised by two experts in running‐related injury using an adapted tool.

**Results:** In total, 2158 records were screened by title and abstract and 41 full‐text records were screened. Two studies, including 18 recreational runners (33.3% female) met the inclusion criteria for this systematic review. Two eligible frameworks were identified ‘the internal locus of injury’ and ‘the injury pathway as experienced by recreational runners’. Both frameworks supported the assertion that the aetiology of running‐related injury is multifactorial, and results from an imbalance of load, capacity, and recovery, which must be self‐regulated by runners in order to avoid running‐related injury.

**Conclusions:** The findings from this systematic review add to the findings from previous research to explain that running‐related injury occurs due to the imbalance of load and capacity, which is mainly regulated by the runner. Clinicians can consider targeting running‐related education through online resources to aid runners' development of skills and knowledge toward self‐regulation of running‐related injury risk and prevention.

## 16 The Role of Podiatrists in Identifying and Addressing Harms From Intravenous Drug Use

Bec Wegener

Cohealth, Fitzroy, Victoria, Australia

Rebecca.wegener@cohealth.org.au

Intravenous (IV) drug use has been a longstanding issue amongst many communities. In recent years there has been a move towards ‘harm reduction’ which is currently run by medical teams of addiction specialist doctors and nurses, as well as peer support and lived experience workers.

Podiatrists' unique and highly qualified position to be able to undertake vascular, neurological and dermatological assessments in a community setting places us in a prime position to be working alongside these specialists.

Current evidence shows IV drug use, particularly in the lower limbs can cause significant and permanent harms including nerve damage, vascular insufficiency and complications, deep vein thrombosis and clots, muscle atrophy, and localised and systemic infections.

Tweaking our existing neurovascular assessments and medical history taking to be able to identify drug harms and upskilling ourselves in this area could place podiatry at the forefront of being able to reduce the frequency and seriousness of chronic lower legs wounds and the complications.

Working for a community health organisation at various sites across Melbourne's inner north west, we have implemented and regularly undertake an amended neurovascular assessment specifically targeted at these client groups which is undertaken as a part of their routine podiatry assessment and treatments. We provide appropriate education, health promotion advice and referrals as required. Secondary to this, by providing a safe and non‐judgemental space we are making moves to reduce stigma and shame within these clients groups. We have seen a high level of engagement from clients and have received positive feedback from service users and internal professionals.

These assessments and templates can serve as a benchmark and a model of care for other organisations in a step towards moving podiatry into the harm reduction space.

## 17 ‘Leaving the Door Open’: Perspectives on Decision‐Making for Non‐Emergency Diabetes‐Related Amputation

Emilee Kim Ming Ong*, Susan Hillier, Carolyn Murray, Ryan Causby

*Allied Health and Human Performance Unit, University of South Australia, North Terrace, Adelaide, South Australia, Australia*

*emilee.ong@mymail.unisa.edu.au

**Background:** Having a lower extremity amputation is a life‐changing decision for people living with a diabetes‐related foot ulcer. Although previous research has described both positive and negative lifestyle and function outcomes of diabetes‐related amputations, limited research has been conducted on the decision‐making processes leading up to the amputation. This study aimed to explore the perspectives of people living with an ulcer or amputation, healthcare practitioners, and expert stakeholders on decision‐making for people with a diabetes‐related foot ulcer who may require a non‐emergency amputation.

**Methods:** A qualitative descriptive study using semi‐structured interviews enabled people to share their thought processes when making decisions for amputation. Twenty‐six participants were interviewed, including nine people with a diabetes‐related foot ulcer or amputation, nine health practitioners, and eight experts located across five countries. There were 13 female and 13 male participants. Thematic analysis was used for data analysis.

**Results:** Four themes described the decision‐making considerations for amputation: ‘Balancing the evidence in decision‐making’, ‘Trust, respect and timing of conversations inform decision‐making’, ‘Tailoring decisions for individual circumstance’ and ‘Reaching the tipping point in decisions for the future’. Work commitments, functional and lifestyle impacts of amputation, the presence of support networks and clinical wound features formed the evidence for a decision for amputation.

**Conclusions:** Understanding quality of life needs ensured that decisions for amputation addressed expectations and lifestyle needs. Living with a diabetes‐related foot ulcer presented daily challenges that pushed people to a tipping point, at which amputation was considered to overcome these hardships and enable them to move on to the next chapter of their life. Consideration of the person in the context of their life, environment and personal needs alongside the pathological factors is warranted when making decisions for amputation. Further research is required to understand how person‐centred factors can be better incorporated alongside objective clinical assessments in decisions for amputation.

## 18 AI‐Powered Detection and Segmentation of Diabetic Foot Ulcers for Accurate Diagnosis and Follow‐Up

Laith Alzubaidi^1,2,^*, Mel Bridges^1^, Yuantong Gu^2^

^1^ *Aptium Company, Brisbane, Queensland, Australia*, ^2^ *School of Mechanical, Medical, and Process Engineering, Queensland University of Technology, Brisbane, Queensland, Australia*

*l.alzubaidi@qut.edu.au

**Background:** Diabetic foot ulcers (DFUs) are a significant complication of diabetes, contributing to severe health impacts and substantial economic burdens globally, with DFU‐related costs estimated at $AUD 1.6 billion annually in Australia alone. Early detection and consistent monitoring of DFUs are critical for improving patient outcomes and reducing advanced complications such as infections and amputations. This study introduces an AI‐driven framework that utilises standard smartphone or camera‐captured foot images for the non‐invasive detection and monitoring of DFUs, offering a practical and accessible solution.

**Methods:** The framework analyses foot images captured using smartphones or standard cameras. The AI system employs Vision Transformers for image classification to detect early signs of ulcers and diabetic foot complications. If abnormalities are detected, the system transitions to a segmentation stage, where the Segformer‐encoder Unet++ model isolates the entire foot and infected regions. Quantitative metrics, such as the ratio of the infected area to the total foot area, are calculated and recorded. Validation was conducted using a dataset comprising 1600 images from patients with diverse demographics, with testing performed under varied lighting and imaging conditions to ensure robustness.

**Results:** The AI‐based framework demonstrated exceptional performance, achieving 98% classification accuracy for detecting early signs of DFUs. The segmentation algorithm, utilising the Segformer‐encoder Unet++ model, achieved a Dice coefficient of 96%, ensuring precise delineation of affected regions. These metrics were validated across a representative dataset, highlighting the system's reliability in diverse real‐world scenarios. The quantitative data enabled reliable progress assessment over multiple visits, simplifying follow‐up care and improving patient engagement in managing their condition.

**Conclusions:** Combining AI‐driven detection and segmentation with smartphone‐based imaging provides a low‐cost, accessible solution for early detection and follow‐up of DFUs. This approach enhances diagnostic precision, supports personalised treatment planning, and empowers both clinicians and patients in resource‐limited settings. By enabling timely intervention, the solution has the potential to improve patient outcomes and reduce healthcare costs significantly. Future work includes broader clinical validation and scalability for widespread adoption.

## 19 Better Feet Better Live, AI‐Powered Thermal Imaging for Early Detection of Diabetic Foot Ulcers

Laith Alzubaidi^1,2,^*, Mel Bridges^1^, Yuantong Gu^2^

^1^ *Aptium Company, Brisbane, Queensland, Australia*, ^2^ *School of Mechanical, Medical, and Process Engineering, Queensland University of Technology, Brisbane, Queensland, Australia*

*l.alzubaidi@qut.edu.au

**Background:** Diabetic foot ulcers (DFUs) are a significant complication of diabetes, significantly impacting patients' health and increasing healthcare costs globally. Early detection is critical to prevent severe outcomes such as amputations, yet conventional diagnostic methods often fail to detect DFUs until advanced stages. Thermal imaging provides a non‐invasive solution, detecting abnormal temperature patterns indicative of early‐stage complications. This study introduces an AI‐powered thermal imaging framework designed to enhance diagnostic precision, improve monitoring, and facilitate timely interventions for DFUs.

**Methods:** The proposed system captures high‐resolution thermal images of patients' feet, highlighting areas with abnormal heat distribution that may indicate poor circulation or inflammation. AI algorithms classify the images into high, low, or normal temperature categories. Another AI model segments the affected regions upon identifying abnormalities and calculating the area for progress tracking. Patient health data, such as blood pressure and glucose levels, are integrated using machine learning algorithms to generate a comprehensive risk profile.

**Results:** The AI system demonstrated exceptional accuracy, achieving 97% classification accuracy for early DFU detection and a Dice coefficient of 96% in segmenting affected regions. The thermal imaging approach detected subtle temperature anomalies, often missed by traditional methods. Quantitative metrics, such as segmented area calculations, provided clinicians with actionable insights to assess ulcer progression, adjust treatment plans, and monitor healing over time.

**Conclusions:** The combination of thermal imaging and AI provides a robust, non‐invasive solution for early detection and monitoring of DFUs. This approach enhances diagnostic accuracy and supports personalised care, reducing the risk of severe complications. Its portable design and user‐friendly interface suit diverse healthcare settings, including underserved and remote areas. The proposed system represents a significant advancement in diabetic foot care, potentially improving patient outcomes and reducing healthcare costs globally. Future work includes expanding clinical validation and scaling the technology for broader adoption.

## 20 The Impact of Podiatric Intervention on the Quality of Life and Pain in Children and Adolescents With Hypermobility

Muhammad Maarj^1,2,^*,Verity Pacey^3^, Louise Tofts^2,3^, Matthew Clapham^4^ Andrea Coda^1,5^

^1^ *School of Health Sciences, College of Health, Medicine and Wellbeing, The University of Newcastle, Ourimbah, Australia*, ^2^ *Narrabeen Sports Medicine Centre, Sydney Academy of Sport, Narrabeen, Australia*, ^3^ *Department of Health Sciences, Macquarie University, Macquarie Park, Australia*, ^4^Hunter Medical Research Institute, New Lambton Heights, Australia, ^5^ *Equity in Health and Wellbeing Research Program, Hunter Medical Research Institute, New Lambton Heights, Australia*

**Background:** The purpose of this study was to evaluate the effect of custom‐made orthotics on pain, health‐related quality of life (HRQoL), function and fatigue in children and adolescents with generalised joint hypermobility (GJH) and lower limb pain.

**Methods:** Fifty‐three children aged 5–18 years were fitted with custom‐made polypropylene orthotics. Visual analogue scale (VAS) assessed lower limb pain severity, Paediatric Quality of Life Inventory assessed HRQoL and fatigue and six‐minute walk test (6 MWT) measured functional endurance at baseline, at 1 and 3 months post‐intervention. A mixed model including a random intercept for participant and a fixed effect for time was used to assess differences in outcomes over time.

**Results:** Fifty‐two children completed the study (mean age 10.6‐years). Children reported significantly reduced pain (mean VAS reduction −27/100, 95% CI: −33, −21), improved HRQoL (mean total improvement 11/100, 95% CI: 7, −15), functional capacity (mean 6MWT improvement 27 m, 95% CI: 18, −36) and fatigue (mean total improvement 13/100, 95% CI: 9, −17) after 1 month of wearing the custom‐made orthotics. From 1 to 3 months there was further statistically but not clinically significant reduction in pain while benefit on other outcomes was maintained.

**Conclusions:** In this study, children with GJH reported reduced lower limb pain, improved HRQoL, functional endurance and fatigue after a month post‐fitting of custom‐made orthotics which was maintained over a 3 month period. Orthotics were well‐tolerated with no serious adverse events reported.

**Keywords:** children; fatigue; generalised joint hypermobility; lower limb pain; orthotics; quality of life

## 21 The Effectiveness and Feasibility of Using Far‐Infra Red Socks to Keep Feet Warm

Malia Ho ^1,^*, Thasvhinni Nasendrana^1^, Daryl Susigan^1^, Gabby Sahota^1^, Alan Illiparambil^1^, Cylie Williams^1^, Alfred Tok^2^, Vitali Lipik^2^, Pengfei He^2^, Natasha Layton^3^

^1^ *School of Primary and Allied Health, Monash University, Frankston, Victoria, Australia*, ^2^ *School of Materials Science and Engineering, Nanyang Technological University, Singapore*, ^3^ *Rehabilitation and Independent Living Research Centre, Monash University, Frankston, Victoria, Australia*

*malia.ho@monash.edu

**Background:** Indian Red Ochre and Haematite are inorganic carbon‐based minerals that emit far‐infrared (FIR) rays. When embedded into fabrics, they are effective in providing heat retention in laboratory settings. Socks constructed with these fabrics have the potential to enhance thermo‐regulating properties for feet. These enhanced socks can be classed as assistive products for self‐care activities and participation in self‐care (AS/ISO 9999 Class 9). However, their efficacy is unknown. The primary aim of this project was to investigate if these FIR socks are effective in keeping feet warm and maintaining blood flow in the lower limbs. The secondary aim is to capture the determinants of effective socks from the product design perspectives.

**Methods:** This double‐blinded repeated measures study included 15 participants who were > 65 years old, and have self‐reported ‘cold feet’. Participants attended 3 sessions at 1‐week intervals to test 3 types of socks, one each embedded with Indian Red Ochre, Haematite and one with no mineral additives (control). At each session, participant's Ankle Brachial Index (ABI), Toe pressure and temperature gradient were measured. The sock was donned for 30 min after which the assessments were repeated. The participants were provided with another 2 pairs of socks to use during the week. Finally, feedback on the sock was collected using a purpose‐built questionnaire.

**Results:** There were no significant statistical differences in ABI, toe pressure and temperature gradient readings between socks. Participants did not feel their feet were warmer compared to the control. Participants liked that the socks felt compressive, but commented that the tightness made them hard to don and doff independently. Participants also felt that the socks were not aesthetically pleasing. These factors resulted in participants not wanting to use the socks.

**Conclusions:** As the socks came in one standard size, without ease of donning and comfort, the actual thermal properties of the materials cannot be fully tested. The tightness of the sock may have restricted blood flow and could have impacted their clinical efficacy in keeping feet warm. This study highlighted the importance of assessing efficacy, together with usability. Future studies should consider making these socks in different sizes.

## 22 The Use of 3D Printing Technology to Create Foot Insoles With Customised Densities

Kiara Qinthara ^1,^*, Hoi Kiu Law^1^, Huarui Cheng^1^, Zifan Wang^1^, Yugendran Arul Jothi^1^, Zhanzhi Liu^1^, Yunlong Tang^1^, Malia Ho^2^

^1^ *Faculty of Engineering, Monash University, Clayton, Victoria, Australia*, ^2^ *School of Primary and Allied Health, Monash University, Frankston, Victoria, Australia*

*kqinthara@gmail.com

**Background:** 3D printing is a sustainable way of fabricating insoles. Unlike traditional made insoles where different materials are glued together, fully 3D printed insoles have the potential to accurately incorporate different properties in one device, with very little material wastage. This study focuses on designing and optimising 3D‐printed personalised insoles tailored for specific foot conditions, such as flat feet. The insoles were fabricated using foaming and normal thermoplastic polyurethane (TPU) filaments. Desired mechanical properties were achieved by varying key parameters such as nozzle temperature (190°C–250°C) and infill density (15%–95%). This case study shows the potential of 3D printing technology in producing a complete insole incorporating the desired support and comfort.

**Methods:** The design process utilised foot scanning technology (3D Size Me) to capture foot geometry and pressure distribution (PEDAR) of one test participant with flat feet. Gensole software was used for orthoses design. A Gaussian Process Regression (GPR) model was developed to predict insole hardness based on printing parameters. Multiple prototypes were produced with combinations of 3D print filaments, varying in infill density and material to achieve targeted hardness and flexibility. Testing, including PEDAR pressure measurements and qualitative feedback, was conducted as a technical case study.

**Results:** The insoles demonstrated Shore A hardness ranging from 23 to 87. The combination of foam TPU and normal TPU (10%–30% infill density) achieved optimum hardness for the following foot regions: forefoot (30–50 Shore A), arch (50–60 Shore A), and heel (60–80 Shore A). Case study testing confirmed up to a 30% reduction in peak pressure in high‐pressure regions. Single user feedback included improvements in support and stability during standing.

**Conclusions:** This technical study demonstrates the potential of 3D printing in creating highly customised insoles, where insole density can specifically address the wearer's individual biomechanical needs, such as redistributing pressure and improving comfort for individuals with flat feet. As part of the technical testing process, the findings indicate promising outcomes for personalised insole solutions. Future collaborative work between clinicians and engineers can explore dynamic testing and further optimisation of material blends to enhance pressure redistribution while maintaining support.

## 23 Foot Orthoses for Midfoot Osteoarthritis (FOMO): A Randomised Controlled Trial

Polly Q. X. Lim ^1,^*, Hylton B. Menz^1^, Karl B. Landorf^1^, Michelle R. Kaminski^1,2,3^, Andrew K. Buldt^1^, Ann Vinicombe^1^, Jaryd Bourke^1,3^, Merridy J. Lithgow^1^, Kade L. Paterson^4^, Jill Halstead^5^, Shannon E. Munteanu^1^

^1^ *Discipline of Podiatry, School of Allied Health, Human Services and Sport, La Trobe University, Melbourne, Victoria, Australia*, ^2^ *Department of Podiatry, Monash Health, Melbourne, Victoria, Australia*, ^3^ *School of Primary and Allied Health Care, Monash University, Melbourne, Victoria, Australia*, ^4^ *Department of Physiotherapy, Centre for Health, Exercise and Sports Medicine, School of Health Sciences, The University of Melbourne, Melbourne, Victoria, Australia*, ^5^ *NIHR Leeds Biomedical Research Centre, and Leeds Community Healthcare NHS Trust, University of Leeds, Leeds, UK*

*p.lim@latrobe.edu.au

**Background:** Midfoot osteoarthritis (OA) is a painful and disabling condition. Arch contouring foot orthoses (FOs) have been recommended for midfoot OA, yet there is no high‐quality evidence from randomised controlled trials to support their use. This clinical trial aimed to evaluate the efficacy of arch contouring FOs for midfoot OA.

**Methods:** This was a parallel‐group, randomised controlled superiority trial. From September 2023 to December 2024, 146 community‐dwelling people with painful midfoot OA were randomised to receive either arch contouring FOs (full‐length heat moulded prefabricated arch contouring FOs made of 140 kg/m^3^ single‐density closed‐cell polyurethane foam) or flat sham inserts (full‐length prefabricated flat polyurethane foam of 3 mm thickness in the same colour, density and branding). All participants were fitted with two pairs of their allocated device into the shoes they wore most regularly and received a standardised education package of clinical guideline‐based information and support for OA. Outcome measures were obtained at baseline, 4, 8 and 12 weeks. The primary endpoint for assessing efficacy was 12 weeks and the primary outcome measure was average midfoot pain whilst walking over the last 7 days on an 11‐point numerical rating scale. Secondary outcome measures included function (walking/standing subscale of the Manchester–Oxford Foot Questionnaire), participants' perception of overall treatment effect (self‐reported global rating of change on a 15‐point Likert scale), physical activity (Incidental and Planned Exercise Questionnaire), general health‐related quality of life (Short Form‐12 Version 2.0), use of co‐interventions and adverse events. The trial was registered prior to recruitment beginning with the Australian and New Zealand Clinical Trials Registry (ACTRN12623000953639).

**Results:** The FOMO trial data analysis is in progress—all participants have been recruited and have completed the 12 weeks data collection period. Full results will be available for the conference.

**Conclusion:** This trial evaluated the efficacy of arch contouring FOs for relieving pain and improving function, physical activity and health‐related quality of life in people with midfoot OA. The findings will provide high‐quality evidence as to whether arch contouring FOs are efficacious and will help to inform clinical guidelines about the use of FOs for midfoot OA.

## 24 Curettage as a Solution for Intractable Plantar Warts: A Case Report

Andrew Slessar*

*Peninsula Foot Clinic, Mount Martha, Victoria, Australia*

*andrews@peninsulafootclinic.com.au

**Introduction:** Plantar warts (verruca plantaris) are cutaneous lesions caused by the human papillomavirus (HPV). Conventional treatments such as cryotherapy, salicylic acid, and laser therapy are commonly used, though none have proven universally effective. This case report highlights curettage as an effective surgical option for recalcitrant plantar warts, demonstrating its potential to provide rapid relief and resolution when other treatments fail.

**Case Presentation:** A 43‐year‐old woman presented with a painful area under her left forefoot, describing it as feeling like constantly walking on a stone. Clinical examination revealed a singular, hyperkeratotic lesion with thrombosed capillaries and loss of normal dermatoglyphics. Palpation confirmed the lesion was rough and tender to medial and lateral compression. The patient was diagnosed with a plantar wart. Previous treatment included six sessions of cryotherapy by her general practitioner, over‐ the‐counter salicylic acid products, and three sessions of SWIFT microwave therapy, all without success. She reported significant pain due to the wart, requiring non‐steroidal anti‐inflammatory medication, particularly during her work as a paramedic. Due to persistent pain and failure of previous treatments, the patient underwent curettage of the plantar wart. Follow‐up at 4 weeks and 6 months confirmed full resolution of the lesion, complete pain relief and no recurrence.

**Discussion:** This case report highlights the challenge of managing intractable plantar warts that have failed other treatments. Treatment outcomes vary depending on the type, size and pain level of the wart. A review of the literature reveals that scarring, infection and recurrence are the most commonly cited complications associated with curettage. This emphasises the need for appropriate training and sound clinical decision‐making processes. This case demonstrates that, when performed by skilled podiatrists, curettage can be both effective and practical, suggesting it could be more widely implemented in clinical practice.

**Conclusion:** Curettage is a safe and effective treatment option for patients who have failed other conventional treatments and are experiencing functional limitations. It can provide rapid resolution and improve quality of life in these patients. Further, more recent studies investigating the effectiveness of curettage for plantar warts are needed, as well as research to establish a more standardised technique.

## 25 A Narrative Case Study of an Industry‐Provider Traineeship Model for Career Development in Podiatry

Caroline Robinson^1,^*, Kerrie Evans^2^, Dean Hartley^2^, Craig Page^2^, Rebecca Gilroy^2^, Jaqueline Robinson^2^, Sharon de Niet^2^, Chantelle Annett^3^, Jordan Duffy^2^, James Lee^2^, Chris Annett^3^, Kirsty Van Grinsven^1^

^1^ *Charles Sturt University, Albury, New South Wales, Australia*, ^2^ *Healthia Limited, Bowen Hills, Queensland, Australia*, ^3^ *Future Feet Podiatry, Shepparton, Victoria, Australia*

*corobinson@csu.edu.au

A steady decline in the recruitment of students to podiatry programs over the past 9 years, has created a workforce shortage in Australia. This shortfall of registered podiatrists is impacting service provision, resulting in unfilled positions and heavy workloads for practitioners. A reduction in podiatry service provision and increased waiting times for an appointment, presents a risk to community health and wellbeing.

Ensuring a sustainable podiatry workforce is now a priority. This is particularly critical in rural and regional areas where a higher proportion of people have risk factors, such as diabetes and chronic health conditions. To augment podiatry services, some lower‐risk podiatry tasks can be undertaken by a podiatry assistant if appropriately trained. However, there are few examples of sustainable, allied health business models that employ assistants and limited access to appropriate podiatry assistant training.

The launch of the Undergraduate Certificate (UG Cert) in Foot Health was the outcome of an industry‐university partnership between Charles Sturt University and Healthia Limited, to develop a novel approach to educating podiatry assistants in the workplace. This industry‐provider traineeship model utilises a novel approach which allows a student to combine paid employment in a podiatry practice and work‐integrated learning, with online study and on‐campus study blocks to complement practical skill development.

This presentation will provide a collective insight to the value of this university program to develop podiatry assistants in the workplace, as told by students, podiatry supervisors, industry partners and academic staff. This initiative demonstrates the value of an industry‐based traineeship model that can be adapted to sustainably introduce podiatry assistants within private and public podiatry services.

The UG Cert creates an accessible course for students who are living outside of metropolitan and regional centres in which current podiatry programs are delivered in Australia. This hybrid mode of study increases learning accessibility for a more diverse range of students and has demonstrated success in enabling students to progress to the Bachelor of Podiatric Medicine. Enabling flexibility for students with university study is an important mechanism to address student recruitment barriers in podiatry, address workforce challenges, and improve access to podiatry care.

## 26 Investigating the Experience of New Zealand Podiatrists Providing Podiatry Care to People With Foot Osteoarthritis

Prue Molyneux ^1,^*, Catherine Bowen^2^, Richard Ellis^1^, Keith Rome^1^, Matthew Carroll^1^

^1^ *School of Clinical Sciences, Auckland University of Technology, Auckland, New Zealand*, ^2^ *School of Health Sciences, Faculty of Environmental and Life Sciences, University of Southampton, Southampton, UK*

*prue.molyneux@aut.ac.nz

**Background:** Current osteoarthritis (OA) care is inconsistent with clinical guidelines, particularly in primary care. Foot OA which causes significant impairments is often overlooked compared to knee and hip OA. The absence of clinical guidelines for foot OA underscored its importance as a research priority. Understanding current assessment and management strategies is crucial before designing clinical trials. This study aims to assess New Zealand (NZ) podiatrists' knowledge of foot OA, their assessment practices, and their management strategies.

**Methods:** A qualitative descriptive approach was employed for data collection and analysis. Using purposive sampling, semi‐structured interviews were conducted with 10 NZ registered podiatrists. An interview schedule guided discussions on diagnosing and managing foot OA. Interviews were audio‐recorded, and transcribed verbatim. Reflexive thematic analysis was used to identify key meanings and patterns within the data.

**Results:** Five key themes derived regarding the assessment of foot OA: (1) chief complaint versus incidental finding; (2) obtaining patient history through subjective interviews; (3) targeted objective assessments for foot OA; (4) determining individual biomechanical factors; and (5) further investigations. Four themes were identified relating to the management of foot OA: (1) knowledge and language used to provide education about OA; (2) a trial‐and‐error approach to modifying joint movement; (3) podiatry administered treatments; and (4) referral pathways to other health professionals.

**Conclusions:** NZ podiatrists utilise an analytical approach to assess and manage foot OA, drawing on their clinical experience and knowledge of OA and lower limb function. Their assessment methods align with published evidence and are comparable to practices in Australia and the United Kingdom. Despite the lack of specific guidelines, foot orthoses and footwear prescriptions are consistently used. However, NZ podiatrists often adopt a traditional view of OA, using impairment‐based language, indicating that advancements in foot OA knowledge are not being effectively translated into clinical practice.

**Clinical Significance:** This research will serve as a catalyst for developing clinical assessment criteria, that can be used both clinically and in future research. As the field of foot OA evolves, it is essential to mobilise knowledge to ensure that advancements are effectively translated into clinical practice.

## 27 Development of an Ultrasound Imaging Atlas (AUTUSI Atlas) for Grading Osteoarthritis in the First Metatarsophalangeal Joint

Prue Molyneux ^1,^*, Catherine Bowen^2^, Richard Ellis^1^, Keith Rome^1^, Kate Fitzgerald^3^, Phillip Clark^3^, Jackie L. Whittaker^4^, Charlotte Dando^2^, Richard Gee^3^, Matthew Carroll^1^

^1^ *School of Clinical Sciences, Auckland University of Technology, Auckland, New Zealand*, ^2^S*chool of Health Sciences, Faculty of Environmental and Life Sciences, University of Southampton, Southampton, UK*, ^3^ *Beyond Radiology, Auckland, New Zealand*, ^4^ *Department of Physical Therapy, Faculty of Medicine, The University of British Columbia, Vancouver, British Columbia, Canada*

*prue.molyneux@aut.ac.nz

**Background:** Ultrasound imaging (USI) may play a fundamental role in the earlier detection and assessment of first metatarsophalangeal joint (MTPJ) osteoarthritis (OA) due to its ability to depict tissue‐specific morphological changes before the point of irreversible structural damage. However, the role of USI in diagnosing OA in foot joints has not been clearly defined. The study aimed to develop a semiquantitative USI atlas (the AUTUSI atlas) to grade osteoarthritic change in the first MTPJ and to evaluate the intra‐examiner and inter‐examiner reproducibility of the atlas.

**Methods:** The AUTUSI atlas was developed using an evidence‐based approach, incorporating data from a systematic review, scoping review, Delphi consensus study, and USI reliability study. Ultrasound images were collected from 57 participants, including 30 with radiographically confirmed first MTPJ OA. The AUTUSI atlas facilitates grading of joint effusion, synovial hypertrophy, synovitis, osteophytes, joint space narrowing, and cartilage thickness. Six examiners used the atlas to independently grade 24 ultrasound images in two sessions. Intra‐examiner and inter‐examiner reproducibility were assessed using percentage agreement and Gwet's AC2. Gwet's AC2 values were interpreted using the following cutoffs: < 0 poor; 0.01–0.20 slight; 0.21–0.40 fair; 0.41–0.60 moderate; 0.61–0.80 substantial; 0.81–1.00 almost perfect or 1.00 perfect.

**Results:** The AUTUSI atlas demonstrated almost perfect‐to‐perfect inter‐examiner agreement (percentage agreement ranged from 96% to 100%, and Gwet's AC2 values ranged from 0.81 to 1.00) and moderate‐to‐perfect intra‐examiner agreement (percentage agreement ranged from 67% to 100%, and Gwet's AC2 values ranged from 0.54 to 1.00).

**Conclusions:** The AUTUSI atlas demonstrated excellent intra‐examiner and inter‐examiner reproducibility for evaluating first MTPJ joint effusion, synovial hypertrophy, synovitis, joint space narrowing, osteophytes, and cartilage thickness. The AUTUSI atlas affords an opportunity to detect prognostic markers of OA earlier in the disease cascade and has the potential to advance understanding of the pathological process of first MTPJ OA.

**Clinical Significance:** Early detection of OA before irreversible structural changes occur could provide a critical window for intervention, enhancing the effectiveness of targeted treatments. The AUTUSI atlas has the potential to improve clinical decision‐making, expand healthcare professionals' capabilities, and enhance healthcare provision, access, and outcomes through early disease detection.

## 28 Podiatric Clinical Triage in a Foot and Ankle Orthopaedic Clinic: A Randomised Trial

Tom P. Walsh ^1,^*, Caitlin Swalwell^1^, Greg B. Merlo^2^, Scott C. Wearing^3,4^, Warren Jacob^5^, Darren Doherty^6^, Margaret Vandermost^6^, Simon Platt^7^

^1^ *Research Office, Gold Coast Hospital and Health Service, Southport, Queensland, Australia*, ^2^ *Healthcare Improvement Unit, Clinical Excellence Queensland, Herston, Queensland, Australia*, ^3^ *School of Medicine and Health, Technical University of Munich, Bavaria, Germany*, ^4^Basel Academy for Quality and Research in Medicine, Basel, Switzerland, ^5^ *Mackay Hospital and Health Service, Mackay, Queensland, Australia*, ^6^ *Interdisciplinary Persistent Pain Centre, Gold Coast Hospital and Health Service, Southport, Queensland, Australia*, ^7^ *Department of Orthopaedics and Trauma, Gold Coast Hospital and Health Service, Southport, Queensland, Australia*

*tom.walsh@health.qld.gov.au

**Background:** Hospitals are increasingly utilising allied‐health professionals to provide clinical triage to patients. While these positions are routinely implemented, and several observational studies have reported positive outcomes, the effectiveness of this intervention is rarely tested in a clinical trial. The objectives of this study were to (i) evaluate a podiatry‐led orthopaedic triage service using patient‐reported outcome measures (PROMs) and (ii) determine if it is cost‐effective in terms of incremental cost/quality‐adjusted life years (QALYs).

**Methods:** This pragmatic, randomised, wait‐list‐control trial recruited participants referred to an orthopaedic foot and ankle clinic at a tertiary‐level health service. Participants were randomised to either immediate clinical triage (intervention) or to remain on the waiting‐list (control). The primary outcome measure was change in foot pain as measured by the Manchester‐Oxford Foot and Ankle Questionnaire (MOxFQ) at 6‐months. Core outcome measures for chronic musculoskeletal pain were also measured and cost‐effectiveness calculated.

**Results:** One hundred and forty‐eight participants were recruited, randomising 73 people to the intervention and 75 people to the control. No between‐group differences in pain or demographics were detected at baseline. Foot pain as measured by the MOxFQ improved in both groups at 6‐months follow‐up, but no significant between‐group differences were identified. Significant between‐group differences, however, were detected in the patient global impression of change. Additionally, the intervention resulted in a 30% discharge rate, and is considered cost‐effective, with each QALY gain costing < $30,000 in real‐world scenarios.

**Conclusions:** A clinical triage service has minimal impact on PROMS for foot and ankle pain or chronic musculoskeletal pain, but it is generally well‐received, is cost‐effective, and participants were more likely to report global improvement if they attended. This study suggests the primary benefit of such clinics may not be to reduce pain. The value in these clinics could be in filtering and educating patients, allowing them to self‐manage, or preparing them for multi‐modal pain or surgical interventions.

## 29 Assessments, Diagnostic Criteria, and Outcome Measures for Paediatric Growing Pains and Painful Restless Leg Syndrome: A Scoping Review

Mitchell Smith ^1,^*, Verity Pacey^1,2^, Luke Davies^1,2^, Cylie Williams^1^

^1^ *School of Primary and Allied Health Care, Faculty of Medicine, Nursing and Health Sciences, Monash University, Melbourne, Australia*, ^2^ *Department of Health Sciences, Faculty of Medicine, Health and Human Sciences, Macquarie University, Sydney, Australia*

*mitchell.smith@monash.edu

**Background:** Children commonly report nocturnal leg pain. Families seek advice from health professionals when it is persistent and results in distress and impact on sleep. In the absence of clear underlying disease, this is often clinically labelled as ‘growing pains’. Previous research has questioned a link between growing pains and restless leg syndrome (RLS), particularly as pain is not strictly required for RLS diagnosis but is often reported. Both conditions have limited focused research and a lack of clear guidelines for assessment. To assist the future development of practice recommendations, this review aimed to collate the assessments, diagnostic criteria, and outcome measures reported in studies of children with growing pains and painful RLS.

**Methods:** This research following the Joanna Briggs Institute (JBI) methodology for scoping reviews. Five online databases were searched from inception to October 2024. Included records reported on the use of assessments, diagnostic criteria, or outcome measures in children with growing pains or persistent lower limb pain in the presence of RLS. Data were extracted by two independent reviewers. Assessments and outcome measures were mapped to the domains of the International Classification of Functioning, Disability, and Health: child and youth version (ICF‐CY).

**Results:** Following screening of 19,806 records, 62 records were included. Most were observational cross‐sectional or case‐control designs. Assessments were varied and primarily focused on body functions and pain characteristics rather than activities and participation. These broadly included subjective questionnaires, physical assessment, imaging and laboratory testing. There were 15 unique diagnostic criteria reported for growing pains with limited consistency and sometimes conflict between included items. Outcomes measures were only reported in 8 records and typically measured pain presence and intensity.

**Conclusions:** Assessment and diagnosis of growing pains and persistent pain in the presence of RLS lacks consistency. Outcome measures were seldom used as most records were not designed to measure change over time. Standardised practices for assessment and management of these conditions may benefit clinicians and optimise patient care. These findings will provide the foundation for further research and development of clinical practice recommendations for paediatric chronic lower limb pain.

## 30 Perceptions and Beliefs Relating to Lower Limb Injuries and Prevention in a Sub‐Elite Netball Club in Queensland, Australia: A Qualitative Study

Lauren Anderson ^1,^*, Megan Hunter^1,2^, Matthew Rixon^1^, Aaron Wholohan^1^, Steven Walmsley^1^

^1^ *School of Clinical Sciences, Queensland University of Technology, Brisbane, Queensland, Australia*, ^2^ *The Health Collab, Brisbane, Queensland, Australia*

*laurenanderson02@icloud.com

**Background:** Lower‐limb injuries, particularly to the knee and ankle, are a leading cause of time‐loss and healthcare costs in netball, significantly impacting players' physical and psychological well‐being. While stakeholder‐driven injury prevention programs improve adoption and efficacy, these approaches remain underexplored in sub‐elite netball—a context marked by professional‐level demands but limited resources. This study investigates the perspectives of players, coaches, and support staff on injury mechanisms, prevention strategies, and barriers within a sub‐elite netball club to identify actionable solutions.

**Methods:** Semi‐structured interviews were conducted with 8 players and 4 staff members from a sub‐elite netball club in Brisbane, Australia. Participants were purposively sampled to capture diverse experiences. Interview transcripts were thematically analysed using constant comparative analysis to identify recurring patterns and relationships. Themes were validated through iterative coding and discussion within the research team.

**Results:** Twenty‐two subthemes emerged, grouped into seven key themes: causes of injuries, lower‐limb injuries, impact of injuries, injury management, organisational relationships, injury prevention, and barriers to prevention. These themes highlighted three critical areas: (1) The journey of lower‐limb injuries, shaped by lifestyle and sub‐elite pressures; (2) The player‐staff dynamic, where communication and trust influenced prevention efforts and (3) The need for tailored prevention strategies. Participants expressed limited confidence in existing programs, citing insufficient evidence of their effectiveness and barriers such as time constraints, financial pressures, and unclear implementation guidelines.

**Conclusions:** This study underscores the urgent need for context‐specific, evidence‐based injury prevention programs tailored to the unique demands of sub‐elite netball. Key recommendations include developing individualised, position‐specific interventions and developing collaborations with governing bodies to ensure program scalability, acceptance, and long‐term sustainability. These findings provide a foundation for reducing injury rates and improving player well‐being not only in sub‐elite netball but also potentially in similar high‐injury sports, advancing the translation of research into practice.

## 31 Impact of Implementing an Orthopaedic Stream in a Metropolitan Tertiary High‐Risk Foot Service

K. Hawkins ^1,^*, E. White^1^, A. James^1^, T. Tran^2^, M. R. Kaminski^1,3,4^

^1^ *Department of Podiatry, Monash Health, Melbourne, Victoria, Australia*, ^2^ *Department of Orthopaedics, Monash Health, Melbourne, Victoria, Australia*, ^3^ *School of Primary and Allied Health Care, Monash University, Melbourne, Victoria, Australia*, ^4^ *Discipline of Podiatry, School of Allied Health, Human Services and Sport, La Trobe University, Melbourne, Victoria, Australia*

*Kate.hawkins@monashhealth.org

**Background:** Diabetes‐related foot disease (DFD) is a leading contributor of global disability, hospitalisations, amputations, and healthcare expenditure. Despite the emphasis on limb salvage and revascularisation in many high‐risk foot services (HRFS), the role of surgery to improve foot function and biomechanics in ulcer healing and recurrence is often underappreciated. This study aimed to evaluate the impact of implementing an orthopaedic stream within an established HRFS.

**Methods:** The new model of care was implemented by an advanced practice orthopaedic podiatrist, and included staff training, establishment of an orthopaedic HRF clinic and ward round, collection of patient and service‐related data, referral pathway development, and credentialing documents. We prospectively recruited patients with DFD who could benefit from orthopaedic consultation and/or intervention. Outcome data were compared to an existing foot and ankle orthopaedic cohort. Inferential statistics explored between‐group and within‐group comparisons.

**Results:** Compared to the foot and ankle cohort (*n* = 134), the HRF cohort (*n* = 79) experienced less wait times for their initial consultation (178.8 vs. 19.8 days) and surgical procedures (313.1 vs. 133.6 days) and had fewer outpatient appointments (3.9 vs. 2.4). There were no readmissions post‐surgery in the HRF cohort, compared to 9 (16.7%) in the foot and ankle cohort. The AusTOMs quality of life measure showed statistically and clinically significant improvements in outcomes within the HRF cohort.

**Conclusions:** The HRF orthopaedic model aligns with local and national objectives, emphasising sustainability, innovation, and patient‐centred care. Overall, there were significant improvements in patient‐ and service‐related outcomes within the HRFS.

## 32 Management of Chronic Oedema Promotes Healing and May Be Associated With Elevation of Toe Pressures: Case Studies of Chronic Lower Limb Oedema, Including Wounds and Lymphorrhoea

Sylvia McAra ^1,^*, Monika Samolyk ^2^

^1^ *Leg and Foot Care, Albury/Wodonga, Victoria, Australia*, ^2^ *Regional Wounds Victoria – Hume East, Accredited Lymphoedema Practitioner, Gateway Health, Wangaratta, Victoria, Australia*

*legandfootcare@gmail.com

This case study series features a crossover trial of multifactorial oedema management in a case of an 89 year‐old woman with chronic oedema complicated by severe lymphorrhoea and hard‐to‐heal leg wounds. At presentation, lymphorrhoea was not contained within daily dressings. Much of the epithelial skin from lower legs had been lost due to the inflammatory excoriation of the lymphorrhoea.

Chronic oedema management was prioritised to address the failure of wound healing. This management included prescriptions of exercise, footwear, foot orthotics, compression garments and intermittent sequential compression pump therapy. This multifactorial management was successful, with resolution of lymphorrhoea and healing of the leg and foot wounds. The chronic oedema management was subsequently withdrawn during a period of hospitalisation. The lymphorrhea and circumferential lower leg wounding recurred during this hospitalisation. When the multifactorial management was recommenced, resolution of lymphorrhoea and healing was achieved for a second time.

Toe pressures monitored throughout this case increased when chronic oedema was successfully managed and reduced when the chronic oedema advanced. These changes in toe pressure may be a predictable feature correlating with the effective management of lower leg chronic oedema.

A tabulated summary of cases presenting with chronic lower leg oedema suggests that increased toe pressures are associated with reduced lower leg circumferences at ankle and calf.

The relevance of this case series is to highlight considerations in the management of people with chronic lower leg oedema. This includes the management of high‐risk foot conditions when wound healing optimisation can be critical to foot and lower limb salvage.

The clinical challenges of multidisciplinary management, including choosing appropriate and safe lower limb compression therapy in cases where arterial supply is questionable, are outlined with some recommendations from current, evidence‐based literature.

## 33 Can Podiatrists Make a Difference in Lower Limb Chronic Oedema?

Sylvia McAra^1,^*, Monika Samolyk ^2^

^1^ *Leg and Foot Care, Albury/Wodonga, Victoria, Australia*, ^2^ *Regional Wounds Victoria – Hume East, Accredited Lymphoedema Practitioner, Gateway Health, Wangaratta, Victoria, Australia*

*legandfootcare@gmail.com

Lower leg chronic oedema is a very common and largely preventable source of morbidity, which predisposes to cellulitis and hard‐to‐heal wounds. Cellulitis is a leading preventable cause of emergency presentations.

The lymphatic system is the third element of the circulatory system, along with arteries and veins. A brief overview of normal lymphatic function and anatomy is presented.

Oedema that has been present for > 3 months is classified as chronic oedema/lymphoedema due to the impairment of the lymphatics over this duration.

Volume overload, abdominal or lower extremity surgery, auto‐immune disease, malignancy, large body size, venous disease, ageing, medications and immobility are just some of the associated risk factors. Aetiology is multifactorial, chronic and complex.

Lower limb chronic oedema has implications for wound healing that are relevant to primary care and to high risk foot management. Compression therapy is one of the mainstays of treatment so when is compression safe to apply in arterial disease? Novel clinical perspectives illuminate decision‐making options.

**Assessment considerations**

– Red flags—cellulitis, pedal ischaemia, acute heart failure
– Lymphorrhoea and inflammatory dermatitis
– Peripheral vascular assessment‐ handheld Doppler waveforms, duplex Doppler ultrasound and toe pressures
– Stemmer's sign and pitting tests are indicators of oedema presence. Recent research on the pitting test indicates accurate assessment relies on effective and consistent method.

**Management principles—introducing the ‘SLAM’ acronym**

– **S**kin and wound care recommendations in line with international consensus on wound hygiene
– **L**ymphatic drainage enhancement—deep breathing, positioning, compression garments, intermittent sequential compression pumps, massage
– **A**ctivity—exercise profoundly enhances lymphatic drainage
– **M**ultidisciplinary management and monitoring.

Podiatrists are ideally positioned to provide key contributions, to the multidisciplinary assessment and management of lower limb chronic oedema in the domains of peripheral arterial supply assessment, biomechanics, wound management, dermatology, footwear and exercise.

Pathways to study for accreditation as a lymphoedema practitioner are outlined.

## 34 Renal Podiatry Service‐Outcomes of a 12‐Month Service Trial at Peninsula Health

Anna Couch*, Giulia Oppedisano, Camilla Phillips, Elise Gillies, Georgia Filosa, Laura Jolliffe

*Peninsula Health, Frankston, Victoria, Australia*

*acouch@phcn.vic.gov.au

**Background:** Adults with Chronic Kidney Disease, particularly End Stage Renal Disease (ESRD), managed via dialysis are at increased risk of foot complications. In 2023, the renal podiatry service was developed to service this at risk cohort of consumers. This research aims to determine the impact of the renal podiatry service for consumers with ESRD managed via dialysis.

**Methods:** This was a mixed methods study of consumers who received care via the renal podiatry service at Peninsula Health. Medical file audits were conducted to describe the cohort and outline the frequency and proportion of podiatry intervention provided over a 12 month period. Consumers who received care via the renal podiatry service were invited to participate in a modified renal treatment satisfaction questionnaire. Dialysis consumer perceptions were explored via 15 one‐on‐one interviews. Inductive thematic analysis was used to analyse data about the impact of the service on consumer's lives.

**Results:** In the first 12 months there were 803 occasions of service. The service provided care to an average of 15.4 consumers per week. Of the total service occasions, 24% (192) included treatment of a foot wound or ulcer. Debridement was performed on 490 occasions; 74% (366) skin, 25% (123) soft tissue and 1% (1) bone/cartilage. A total of 64 consumers completed the satisfaction questionnaire, 92% (59) participants were very satisfied with the podiatry care they had received whilst on dialysis and found it very convenient. Three key themes were generated through analysis of the one‐on‐one interviews, including (i) the burden of time, (ii) responsive care—‘the whole kit and caboodle’ and (iii) visible financial flexibility.

**Conclusions:** Results highlight the positive impact the service has had on alleviating barriers of access to foot care. This study identified the invisible mental load of managing appointments for those with chronic ill health.

## 35 Hallux Valgus: Footwear, Genetics, or Both?

Hylton B. Menz*

La Trobe University, Melbourne, Victoria, Australia

**Background:** Hallux valgus (HV) is common and is associated with decreased health‐related quality of life, footwear difficulties, impaired balance and increased risk of falls. However, the cause of HV is still poorly understood. In this presentation, the relative contribution of footwear and genetics will be considered.

**Methods:** This presentation draws on several investigations which have examined associations between HV and compressive footwear (the Clinical Assessment Study of the Foot), heritability using pedigree charts (the Framingham Heart Study), genome‐wide association studies (the Framingham Heart Study, the Genetics of Generalized Osteoarthritis Study, the Johnston County Osteoarthritis Project, and the Osteoarthritis Initiative) and twin studies (the Australian Twin Registry, the Korean Healthy Twin Study).

**Results:** HV is associated with compressive footwear, with a dose‐response relationship observed between HV and increasing toe‐box compression, and evidence that 18 out of 100 cases are potentially preventable. HV is moderately to highly heritable (45%–63%), and may be genetic, although no specific gene or combination of genes has yet been identified. The association with footwear is stronger in identical (monozygotic) twins, which suggests that footwear may be a shared environmental risk factor.

**Conclusions:** HV is related to both footwear and genetics. However, given that footwear is a more modifiable risk factor, clinicians should continue to advise against the use of compressive footwear where possible.

## 36 Foot Osteoarthritis Research: A Bibliometric Analysis

Hylton B. Menz*

*La Trobe University, Melbourne, Victoria, Australia*

**Background:** Foot osteoarthritis (OA) is a common and disabling condition that has been relatively neglected by researchers, despite the finding that its prevalence is similar to that of knee OA and more than double that of hip OA. The purpose of this study was to conduct a bibliographic analysis to understand who is performing foot OA research, what type of research is being conducted and where, and how foot OA research is funded.

**Methods:** The Scopus database search was conducted to identify all non‐surgical foot OA articles published in English up until December 2023. Bibliometric analysis was performed using an open‐source tool based on the R language. Citations, journals, authors, institutions, and countries were described. Publications were manually categorised according to research type and funding source.

**Results:** The search strategy yielded 121 eligible articles, which received a total of 4531 citations and were published by 372 authors in 55 journals. The highest publication output occurred in the past decade. The most frequent journals were *Arthritis Care & Research*, *Osteoarthritis and Cartilage*, *Journal of the American Podiatric Medical Association*, *Annals of the Rheumatic Diseases*, *Foot & Ankle International* and *Journal of Foot and Ankle Research*. The most published institutions were Keele University, La Trobe University, the University of Leeds, the University of Melbourne and the University of North Carolina. Of the 106 articles that could be classified, most were focused on aetiology (*n* = 52, 49%), followed by evaluation of treatments and therapeutic interventions (*n* = 28, 26%), detection, screening and diagnosis (*n* = 17, 16%), health and social care services research (*n* = 6, 6%) and underpinning research (3, 3%). Fifty‐one articles (42%) reported no research funding.

**Conclusions:** Foot OA research has increased significantly in the past decade. There is a need, however, to improve our understanding of the condition and to address the relatively small number of clinical trials that have been conducted.

## 37 Evaluation of a Multi‐Camera Photogrammetry‐Based 3D Scanner for Capture of Geometry of the Foot for Design of Orthoses

Joshua Taylor^1^, Alexander J. Terrill ^1,2,3,^*, Aaron Wholohan^1^, Renee Nightingale^2,3^, Sean K. Powell^2,3,4^, Maria A. Woodruff^2,3,4^

^1^ *School of Clinical Sciences, Queensland University of Technology, Brisbane, Queensland, Australia*, ^2^ *School of Mechanical, Medical, and Process Engineering, Queensland University of Technology, Brisbane, Queensland, Australia*, ^3^ *Centre for Biomedical Technologies, Queensland University of Technology, Brisbane, Queensland, Australia*, ^4^ *Aptium.ai, Brisbane, Queensland, Australia*

*alexander.terrill@hdr.qut.edu.au

**Background:** Three‐dimensional scanning has revolutionised the capture of foot geometry for the digital production of personalised orthoses, providing time and cost savings. Different 3D scanning techniques vary in accuracy and speed based on their underlying technology. One such technique, photogrammetry, uses several photos of the foot to generate a 3D model. While not currently widely used by podiatrists, multi‐camera photogrammetry allows for ‘instantaneous’ scanning. This study compares accuracy, scanning time, and model generation times of several 3D scanners, including multi‐camera photogrammetry, to evaluate their clinical suitability.

**Methods:** The plantar surface of the left feet of 20 healthy participants were scanned in a non‐weight bearing subtalar joint neutral position. A high‐accuracy industrial 3D scanner (Artec Spider) was used as a control for evaluating the accuracy of clinical scanners, including an Apple iPad 6 with Structure Sensor (iPad), an Envisic Veriscan laser scanner (laser), and a prototype Aptium.ai multi‐camera photogrammetry scanner (multiscan). The root‐mean‐square‐error (RMSE) between 3D mesh of the control scanner and each of the study scanners was computed using CloudCompare software, with a smaller RMSE indicating greater accuracy. The corresponding scan capture and 3D model generation times were also compared.

**Results:** The mean RMSE over all participants was 0.36 ± 0.11 mm for the iPad, 0.66 ± 0.18 mm for the laser, and 0.26 ± 0.07 mm for the multiscan, with corresponding scan times of 60 ± 11, 24 ± 1, and < 1 s, respectively. The mean 3D model generation times were 29 ± 4 s for the iPad, 49 ± 6 s for the laser, and 414 ± 48 s for photogrammetry. The Artec Spider took an average of 301 ± 78 s for 3D scan capture and 600 ± 175 s for 3D model generation.

**Conclusions:** All three scanners demonstrate accuracy suitable for personalised foot orthoses design. The scan capture times were also < 1 min for all scanners, however, the nearly instant scanning of the multiscan prototype offers potential benefits when scanning people who cannot remain still, such as children and individuals with hyperkinetic movement disorders. It can be expected that hardware and software advances would lower the 3D model generation times for all scanners, further improving the tools available for podiatrists.

## 38 Evaluation of Personalised 3D Printed Functionally Graded Metamaterial Foot Orthoses With an Offloading Boot to Reduce Plantar Pressure

Alexander J. Terrill ^1,2,3,^*, Edmund I. Pickering^1,2^, Sean K. Powell^1,2,4^, Peter A. Lazzarini^5,6^, Maria A. Woodruff^1,2,4^, David W. Holmes^1,2^

^1^ *School of Mechanical, Medical, and Process Engineering, Queensland University of Technology, Brisbane, Queensland, Australia*, ^2^ *Centre for Biomedical Technologies, Queensland University of Technology, Brisbane, Queensland, Australia*, ^3^ *School of Clinical Sciences, Queensland University of Technology, Brisbane, Queensland, Australia*, ^4^ *Aptium.ai, Brisbane, Queensland, Australia*, ^5^ *School of Public Health and Social Work, Queensland University of Technology, Kelvin Grove, Queensland, Australia*, ^6^ *Allied Health Research Collaborative, Metro North Hospital and Health Service, Brisbane, Queensland, Australia*

*alexander.terrill@hdr.qut.edu.au

**Background:** 3D printing enables rapid and low‐cost manufacture of personalised foot orthoses and opens new opportunities to enhance their mechanical function. One opportunity is the use of metamaterials, which are a class of structure with complex internal architectures that enables precise control of mechanical properties including stiffness. Functionally graded metamaterials (FGMs) have an internal architecture that varies through the structure, leading to mechanical properties that vary smoothly and continuously. FGMs could be leveraged to improve pressure redistribution and may be of particular benefit in designing orthoses for offloading treatment of diabetes‐related foot ulceration (DFU) when used with guideline‐recommended boots. By varying stiffness across an orthosis, softer support can be placed under higher pressure areas, and stiffer support under lower pressure areas, potentially improving offloading effectiveness. While FGMs allow for tailorable orthosis stiffness to improve offloading, there is little known about how to optimally distribute stiffness. To address this need, we developed a novel workflow to produce FGM orthoses using fused filament 3D printing and compared three algorithms that design orthosis stiffness based on plantar pressure data.

**Methods:** Fourteen healthy participants were recruited for a within‐subjects immediate‐effects crossover trial. For each participant, three flat and one contoured FGM orthoses were 3D printed, with the stiffness of each insole tuned by a different automated algorithm. Comparators included a contoured ethylene‐vinyl acetate orthosis and a flat prefabricated orthosis. Each insole was worn within an ankle‐high offloading boot, and changes in plantar pressure were measured. This study was prospectively registered with Australian New Zealand Clinical Trials Register (ACTRN12624001175561).

**Results:** A novel automated workflow was developed to design and 3D print personalised FGM offloading orthoses based upon plantar pressures. For each participant, peak plantar pressure and offloading effectiveness was determined. The orthosis design algorithms were compared, and their effectiveness will be presented.

**Conclusions:** This research provides guidance on automatic design algorithms for plantar pressure offloading in 3D printed FGM orthoses. We provide insight on how mechanical properties affect pressure redistribution and the most suitable design algorithms for FGM orthoses. The findings of this study with healthy participants will inform future studies recruiting participants with DFU.

## 39 Plantar Heel Pain: Five Updates You Need to Know

Karl B. Landorf*

*Discipline of Podiatry, La Trobe University, Victoria, Australia*

*k.landorf@latrobe.edu.au

**ABSTRACT**

Knowledge never remains stagnant, particularly in healthcare. Accordingly, new information frequently becomes available, and this is the case with plantar heel pain, a highly prevalent and often disabling condition. This presentation will summarise five important issues relating to plantar heel pain where knowledge has evolved over the last few years. These issues include: (i) prevalence, (ii) prognosis, (iii) risk factors, (iv) tissue involvement other than the plantar fascia and naming of the condition and (v) treatment. This information will provide clinicians with an important update for plantar heel pain, so they can provide up‐to‐date information and realistic expectations to patients that they treat.

## 40 Student Perspectives on Marketing the Podiatry Profession and Course Promotion: A Mixed Methods Study

Michelle R. Kaminski ^1,2,3,^*, Caroline Robinson^4^, Glen A. Whittaker^1^, Malia Ho^3^, Daniel R. Bonanno^1^, Shannon E. Munteanu^1^, Mollie Dollinger^5^, Sia Kazantzis^6^, Xia Li^7^, Ryan S. Causby^8^, Mike Frecklington^9^, Steven Walmsley^10^, Vivienne Chuter^11^, Sarah L. Casey^12^, Burke Hugo^13^, Matthew Cotchett^1^

^1^ *Discipline of Podiatry, School of Allied Health, Human Services and Sport, La Trobe University, Melbourne, Victoria, Australia*, ^2^ *Department of Podiatry, Monash Health, Melbourne, Victoria, Australia*, ^3^ *School of Primary and Allied Health Care, Monash University, Melbourne, Victoria, Australia*, ^4^ *School of Allied Health, Exercise and Sports Sciences, Charles Sturt University, Albury, New South Wales, Australia*, ^5^ *Faculty of Health Sciences, Curtin University, Bentley, Western Australia, Australia*, ^6^ *Department of Physiotherapy, Melbourne School of Health Sciences, The University of Melbourne, Melbourne, Victoria, Australia*, ^7^ *Mathematics and Statistics, School of Computing, Engineering and Mathematical Science, La Trobe University, Melbourne, Victoria, Australia*, ^8^ *Allied Health and Human Performance Unit, University of South Australia, Adelaide, South Australia, Australia*, ^9^ *Department of Podiatry, School of Clinical Sciences, Faculty of Health and Environmental Sciences, Auckland University of Technology, Auckland, New Zealand*, ^10^ *Discipline of Podiatry, School of Clinical Sciences, Faculty of Health, Queensland University of Technology, Brisbane, Queensland, Australia*, ^11^ *School of Health Science, Western Sydney University, Campbelltown, New South Wales, Australia*, ^12^ *Discipline of Podiatry, School of Health Sciences, Western Sydney University, Campbelltown, New South Wales, Australia*, ^13^ *Division of Podiatric Medicine and Surgery, School of Allied Health, University of Western Australia, Perth, Western Australia, Australia*

*m.kaminski@latrobe.edu.au

**Background:** The decline in podiatry student enrolments at universities across Australia and New Zealand presents a significant workforce challenge, threatening the profession's sustainability and community health outcomes. Recent data highlights a limited understanding of the podiatry profession among allied health students and identifies actions to address negative stereotypes and to build knowledge of the profession's scope of practice, career opportunities, job prospects, and earning potential. As part of a broader research initiative, this study explored student perspectives on marketing the podiatry profession to increase student enrolments.

**Methods:** A convergent mixed methods study design was employed. Participants included students enrolled in (i) podiatry and (ii) relevant ‘non‐podiatry’ health, sport or science programs at nine Australian universities and one New Zealand university. Data were collected via an online survey (278 podiatry students and 553 non‐podiatry students responding) and two online workshops with nine first‐year podiatry students. Quantitative data were analysed using descriptive statistics and regression models, while qualitative data underwent thematic analysis by three independent assessors.

**Results:** Findings revealed that over 40% of podiatry students had initial concerns about their course, and 34.2% had considered leaving. Instagram and Facebook were the two most highly rated social media platforms for sourcing information about careers and courses available. Four key themes emerged relevant to marketing strategies to increase student enrolments: (i) enhance the visibility, perception, and advocacy of podiatry; (ii) emphasise holistic and diverse practice in podiatry; (iii) enable early exposure and experience of podiatry practice; (iv) improve course entry pathways and flexibility.

**Conclusions:** An evidence‐based marketing approach is required to enhance the visibility and appeal of podiatry as a career. Strategies should focus on addressing misconceptions, expanding promotional efforts to broader audiences, leveraging relevant media platforms, reducing barriers to entry, and improving study flexibility. Strengthening enrolments and reducing attrition are fundamental to ensuring the long‐term sustainability and growth of the podiatry profession in Australia and New Zealand.

## 41 Motivators and Barriers for Studying Podiatry in Australia and New Zealand: A Mixed Methods Study

Michelle R. Kaminski ^1,2,3,^*, Glen A. Whittaker^1^, Caroline Robinson^4^, Matthew Cotchett^1^, Malia Ho^3^, Shannon E. Munteanu^1^, Mollie Dollinger^5^, Sia Kazantzis^6^, Xia Li^7^, Ryan S. Causby^8^, Mike Frecklington^9^, Steven Walmsley^10^, Vivienne Chuter^11^, Sarah L. Casey^12^, Burke Hugo^13^, Daniel R. Bonanno^1^

^1^ *Discipline of Podiatry, School of Allied Health, Human Services and Sport, La Trobe University, Melbourne, Victoria, Australia*, ^2^ *Department of Podiatry, Monash Health, Melbourne, Victoria, Australia*, ^3^ *School of Primary and Allied Health Care, Monash University, Melbourne, Victoria, Australia*, ^4^ *School of Allied Health, Exercise and Sports Sciences, Charles Sturt University, Albury, New South Wales, Australia*, ^5^ *Faculty of Health Sciences, Curtin University, Bentley, Western Australia, Australia*, ^6^ *Department of Physiotherapy, Melbourne School of Health Sciences, The University of Melbourne, Melbourne, Victoria, Australia*, ^7^ *Mathematics and Statistics, School of Computing, Engineering and Mathematical Science, La Trobe University, Melbourne, Victoria, Australia*, ^8^ *Allied Health and Human Performance Unit, University of South Australia, Adelaide, South Australia, Australia*, ^9^ *Department of Podiatry, School of Clinical Sciences, Faculty of Health and Environmental Sciences, Auckland University of Technology, Auckland, New Zealand*, ^10^ *Discipline of Podiatry, School of Clinical Sciences, Faculty of Health, Queensland University of Technology, Brisbane, Queensland, Australia*, ^11^ *School of Health Science, Western Sydney University, Campbelltown, New South Wales, Australia*, ^12^ *Discipline of Podiatry, School of Health Sciences, Western Sydney University, Campbelltown, New South Wales, Australia*, ^13^ *Division of Podiatric Medicine and Surgery, School of Allied Health, University of Western Australia, Perth, Western Australia, Australia*

*m.kaminski@latrobe.edu.au

**Background:** Podiatry enrolments at Australian and New Zealand universities have decreased by 17.3% since 2015, which threatens the profession's sustainability and the health and wellbeing of Australian and New Zealand people and communities. Reasons for this decline remain unclear due to insufficient evidence on factors influencing career choices. The overarching aim of this study was to identify motivators and barriers for studying podiatry in Australia and New Zealand.

**Methods:** This study used a convergent mixed methods design. Students enrolled in (i) podiatry and (ii) relevant non‐podiatry health, sport or science programs at nine Australian and one New Zealand university, were invited to participate in an online survey. First‐year podiatry students were also invited to participate in an online workshop. Quantitative data were analysed using descriptive statistics and linear/logistic regression models. Three independent assessors used inductive thematic analysis for the qualitative data.

**Results:** Overall, 278 podiatry students (mean age 24.9 ± 8.5 years, 65.1% female) and 553 non‐podiatry students (mean age 24.8 ± 8.2 years, 75.4% female; 32.2% from physiotherapy and 29.1% from occupational therapy) responded to the survey. Interest in a health‐related career, wanting to make a difference to people's health, and opportunity to care for people from different backgrounds/age groups were key motivating factors among podiatry students. Barriers to studying podiatry were encountered by 28.1% of podiatry students. Thematic analysis identified seven themes concerning career choice, which are as follows: (i) awareness of profession and scope of practice; (ii) stereotypes and negative perceptions of the profession; (iii) awareness of career pathways; (iv) job prospects and earning potential; (v) working with people and building relationships; (vi) podiatry is not the first preference and (vii) barriers which limit student enrolment.

**Conclusions:** There are a variety of factors that motivate and influence students to study podiatry, however, altruistic reasons are most highly rated. Allied health students have limited understanding of the scope of practice and career opportunities in podiatry. Additionally, the podiatry profession often faces negative stereotypes. Further work is required to reverse the negative stereotypes and perceptions of podiatry and build knowledge of the profession's scope of practice, career pathways/opportunities, job prospects and earning potential.

## 42 A Prospective Cost‐Minimisation Analysis of Fixation Used in Distal Metatarsal Chevron Osteotomy (Protocol)

Ryan James^1,^*, Mark Gilheany^1^, Matthew Cotchett^2^

^1^ *Australasian College of Podiatric Surgery, Melbourne, Australia*, ^2^ *Department of Physiotherapy, Podiatry, Prosthetics and Orthotics, Discipline of Podiatry, College of Science, Health and Engineering, La Trobe University, Melbourne, Victoria, Australia*

*rj.podiatry@gmail.com

**Background:** The chevron distal first metatarsal osteotomy is a well‐established surgical technique with or without additional Akin osteotomy for the treatment of Hallux Abducto Valgus deformity. Over the years, various modifications and fixation techniques have been utilised for the chevron component of the procedure. More recently, the minimally invasive Chevron Akin (MICA) procedure has also gained popularity. However, there is limited research comparing these techniques. In addition to comparison of patient focused outcomes and complication rates cost comparison is an important indicator in considering adoption of new techniques. This project seeks to quantify the relative costs of different types of fixations for chevron‐type osteotomies utilising a cost‐minimisation analysis.

**Methods:** This prospective cost minimisation analysis follows the guidelines of the Conduct, Methodological Practices, and Reporting of Cost‐effectiveness Analysis, Second Panel on Cost‐Effectiveness in Health and Medicine. We will survey experienced foot and ankle surgeons who perform distal metatarsal chevron osteotomies to assess the direct costs associated with different fixation methods, including k‐wires, screws, or other constructs. The analysis will consider both healthcare and societal perspectives, encompassing costs paid by third‐party payers and patients. Confidence intervals, hypothesis testing, and cost ratios will be used for data analysis.

**Results:** The study will provide mean cost estimates with 95% confidence intervals for each fixation construct. A cost ratio analysis will be performed to assess cost differences between various fixation methods. Initial analysis suggests that for the same procedure, in excess of $2000 can be saved by choosing certain fixation methods without compromising clinical outcomes. Potential economic factors surveyed include the use of modifications to the Austin technique, types of fixation, and complications.

**Conclusions:** While podiatrists may not perform surgical procedures themselves, understanding the health economics of these interventions is essential for making ethical and informed referral decisions. This cost‐minimisation analysis provides critical insights into the economic implications of various fixation constructs used in distal metatarsal chevron osteotomies. By quantifying direct costs and exploring cost‐effectiveness, the study highlights the importance of balancing clinical outcomes with financial sustainability. Empowering podiatrists with this knowledge ensures referrals are not only guided by clinical efficacy but also by the ethical responsibility to advocate for the least costly method that achieves the same patient outcomes. These findings have the potential to shape referral practices, influence policy‐making, and promote cost‐conscious, patient‐centred care in the treatment of hallux valgus and related conditions.

## 43 Middle Phalangectomy for the Correction of Macrodactyly of the Second Digit in an Adult Patient: A Case Report

Ryan James*, Abdel Kak, Haydar Ozcan

*Australasian College of Podiatric Surgeons, Melbourne, Victoria, Australia*

*rj.podiatry@gmail.com

**Background:** Macrodactyly, a rare congenital anomaly characterised by localised gigantism of one or more digits, presents significant challenges in therapeutic management due to the absence of established guidelines. This case report explores the surgical management of an adult patient with macrodactyly of the second digit, addressing discomfort, footwear issues, and psychological distress. The study adapts techniques from prior paediatric cases to suit adult care needs, particularly focusing on middle phalangectomy as a feasible treatment option for isolated macrodactyly.

**Methods:** Case report does not have a method.

**Results:** Clinical examination and radiographic assessments confirmed primary macrodactyly affecting the patient's left second toe. The surgical intervention involved middle phalangectomy, debulking of fibrofatty tissue, joint capsule, and tendon repair, along with a Winograd wedge resection procedure. Postoperative care included pain management and wound dressing. Despite encountering delayed wound healing and mild necrotic changes, the patient reported manageable pain levels and expressed satisfaction with the cosmetic outcome and improved footwear options at the 6‐month follow‐up.

**Conclusions:** The management of rare congenital deformities with limited or absent clinical guidelines necessitates an adaptive approach, drawing upon principles from analogous cases in the literature. This original case report not only demonstrates the application of these principles but also serves as a valuable educational resource for podiatrists. By raising awareness and fostering understanding of these uncommon conditions, it provides a framework for clinicians to navigate the complexities of similar cases in their practice.

## 44 Charcot Foot Health Literacy Among Individuals With Diabetic Peripheral Neuropathy in Scotland

Benjamin Bullen*

*University of Galway, Galway, County Galway, Ireland*

*benjamin.bullen@universityofgalway.ie

**Background:** Charcot foot is a potentially devastating complication of diabetic peripheral neuropathy. The frequently asymptomatic nature of this condition may lead to delayed recognition, diagnosis and pressure relief, associated with a characteristic ‘rocker bottom’ foot deformity. Deformity may, in turn, precipitate diabetes foot ulceration, infection and, ultimately, lower extremity amputation. Podiatric diabetes self‐management education and support strategies have historically focused on ulceration and amputation prevention, reserving Charcot foot information for those with ‘Active’ disease.

**Methods:** Specific traits and social support required to recognise and respond to early Charcot ‘danger signs’ were defined as ‘Charcot foot health literacy’. A resultant ‘Charcot foot health literacy conundrum’ was proposed, as individuals with diabetic peripheral neuropathy, ‘At‐risk’ of Charcot foot, were not informed of their risk status nor did they receive targeted education or support. The research hypothesis was that individuals with Charcot foot experience, classified as ‘In Remission’, developed specific Charcot foot health literacy traits through exposure to podiatric diabetes self‐management education and support and reflection on past experience. The Comparing Charcot foot Health literacy between ‘At‐risk’ and ‘In Remission’ groups (CCHAIR Study) compared general, multidimensional health literacy and ‘Knowledge’, ‘Understanding’ and ‘Contextualisation’ Charcot foot health literacy skills between people with and without Charcot foot experience, with the Health Literacy Questionnaire and a CCHAIR Study Qualitative Questionnaire, respectively.

**Results:** Study findings confirmed ‘At‐risk’ individuals (*n* = 29) lacked Charcot foot knowledge, compared with those ‘In Remission’ (*n* = 34; *p* < 0.000). All participants (*n* = 63) more readily associated this condition with late‐stage complications, rather than early inflammatory signs. Charcot foot experience was associated with active self‐management (*p* = 0.034) and those ‘In Remission’ remained engaged with specialist podiatry and/or multidisciplinary services. Findings were both statistically and clinically significant.

**Conclusions:** Future diabetes self‐management education and support approaches should target all individuals with diabetic peripheral neuropathy, focusing on prompt self‐recognition of, and self‐referral in the event of, early Charcot foot ‘danger signs’.

## 45 Exploring the Impact of Material Aid Access to Improve Foot Health: Addressing Health Inequity, Delivering Social Justice, and Empowering Individuals

Anthony Lewis^1,^*, Shan Bergin^1,2^, Anna Stybowski^1,3^, James Gerrard^1,4^

^1^ *Footscape, Naarm (Melbourne), Wurundjeri Country, Victoria, Australia*, ^2^ *Discipline of Podiatry, School of Allied Health, Human Services and Sport, La Trobe University, Bundoora, Wurundjeri Country, Victoria, Australia*, ^3^ *Danila Dilba Health Service, Garramilla (Darwin), Larrakia Country, Northern Territory, Australia*, ^4^ *Central Australian Aboriginal Congress, Mparntwe (Alice Springs), Arrernte Country, Northern Territory, Australia*

**Background:** Footscape was established in 2009 and is a registered charity assisting people disadvantaged by systems who are living with foot concerns. Organisation projects include provision of podiatrist dispensed materials (footwear, socks, orthotic devices, and foot care kits) for people experiencing homelessness, financially disadvantaged children, asylum seekers, First Nations Peoples, and survivors of domestic violence. During October 2024 Footscape reached 100,000 items of material aid distributed [1].

**Methods:** Footscape material aid previously featured in original research exploring foot health support for individuals experiencing homelessness [2]. In 2024 Footscape received a grant from the Collier Charitable Fund [3] to conduct an extended material aid project evaluation. Being well suited to addressing the complexity of public health problems and their solutions [4], mixed methods will be used in this evaluative study to provide comprehensive multi‐level analysis [5] of material aid outputs and the impacts access to them has on foot health and overall wellbeing at both organisational and individual levels. Ethical approval will be obtained from relevant Human Research Ethics Committee(s) and all participants will provide informed consent prior to participating in the study.

**Results:** Anecdotal feedback as part of this work's needs analysis and grant application indicates that material aid access improves the ability of project affiliate organisations to support individuals. ‘Footscape's generous provision of high‐quality second‐hand shoes has greatly improved our ability to support these clients as they work to achieve their goals’. ‘Foot Care Kits… prompt conversation and get people actively involved in learning self‐foot care’. ‘Where there is often a large level of mistrust, for good reason, of the dominant western health system, being able to open up a positive yarning experience with physical props (sharing footwear) builds trust and rapport which is the baseline for any meaningful work’.

**Conclusions:** Conclusions will generate new and diverse insights [5] and enable depth and breadth of understanding [4] in this field. The significance of this study lies in its positioning of podiatry in addressing health inequity, delivering social justice, and empowering individuals, and in providing a whole of profession involvement in this pursuit through opportunity to be involved in the charity's work.

**References**

1. Footscape, “Footscape Homepage – Material Aid,” (2024), https://footscape.com.au.
2. R. Ogrin, M. A. Rushford, J. Fallon, R. Mannix, B. Quinn, and A. Lewis, “Describing the Development and Implementation of a Novel Collaborative Multidisciplinary Approach to Deliver Foot Health Supports for Individuals Experiencing Homelessness and Its Outcomes,” *PLoS One* 19, no. 4 (2024): e0302572, 10.1371/journal.pone.0302572.
3. Collier Charitable Fund, “Collier Charitable Fund – Funding Approach,” (2024), https://www.colliercharitable.org/.
4. L. A. Palinkas, S. J. Mendon, and A. B. Hamilton, “Innovations in Mixed Methods Evaluations,” *Annual Review of Public Health* 40, no. 1 (2019): 423–442, 10.1146/annurev‐publhealth‐040218‐044215.
5. M. Bamberger and L. Mabry, *RealWorld Evaluation: Working Under Budget, Time, Data, and Political Constraints* (Sage Publications, 2019).

## 46 Making Your Website Accessible to the 1 in 6 Australians Who Live With a Disability

Cylie M. Williams*, Willow Metcalf

*Monash University, Frankston, Australia*

*cylie.williams@monash.edu

There are approximately 5.5 million (or 1 in 6 Australians) who live with a disability. Disability rates vary over the life span, as does the structural support required for access to health services.

The majority of Australians use the internet to gather information and find out about health services. A recent audit of podiatry websites using the standards from the international web accessibility initiative (W3C WAI), identified substantial issues in many websites relating to operability, perceptibility and understandability. Website accessibility issues can lead to people with disability having difficulty or being completely restricted from accessing health services and information. These barriers can contribute to lower health outcomes and impact health literacy amongst people with disability.

Many of the issues can be easily fixed. This practical workshop will introduce how to identify any accessibility issues within your website, why it's important to fix these and provide tools and tips for you to improve accessibility moving forward.

## 47 Patient and Clinician Perceptions of the Psychosocial Challenges Associated With Diabetes Related Foot Ulcers

Shan Bergin ^1^^,^*, Anita Rasppovic^1,2^

^1^ *Disicpline of Podiatry, School of Allied Health, Human Services and Sport, La Trobe University, Bundoora, Victoria, Australia*, ^2^ *School of Psychology and Public Health, La Trobe University, Wodonga, Victoria, Australia*

**Background:** Outcomes related to the clinical management of diabetes related foot ulcers (DFU) are well documented, however the role of psychosocial impacts on these outcomes is less well considered. The aim of this study was to conduct a qualitative exploration of patient and podiatrist perceptions of psychosocial impacts of DFU.

**Methods:** Semi structured interviews were conducted with patients and podiatrists. Adult patients with DFU and podiatrists with a minimum of 5 years' experience working in a high‐risk foot setting participated. Open ended questions were used to elicit detailed patient and podiatrist perceptions regarding psychosocial aspects of DFU including the lived experience, challenges associated with treatment and strategies adopted to overcome challenges and better address psychosocial elements of care. Interviews were audio recorded and transcribed. Data was analysed thematically.

**Results:** Eleven podiatrists and 10 patients were interviewed. Multiple themes were identified including behavioural responses and coping mechanisms, psychosocial considerations and improved management of DFU. Patients consistently reported negative feelings associated with DFU, including fear, anxiety and distress. Podiatrists identified that factors including cultural diversity and health literacy can influence the lived experience. Both adaptive and maladaptive coping responses were reported by patients. Maladaptive responses included avoidance, disengagement or deflection of responsibility to others. Some patients adopted a positive attitude to treatment however patients and podiatrists reported difficulty with sustaining that positivity over time. Patients and podiatrists recognised the importance of a trusted therapeutic alliance, empathy and patient empowerment as components of clinical care, however podiatrists reported that time, resources and scope of practice concerns impacted their ability to adequately address psychosocial aspects of care. Patients reported the importance of being ‘heard’ and included in decisions regarding their care as well as improvements to financial and social support. Podiatrists reported a need for improved training and education around psychosocial support, implementation of screening tools and improved access to psychological services.

**Conclusions:** Psychosocial impacts of DFU are an important determinate for clinical outcomes and must be incorporated into future research, clinical practice guidelines and professional development to support truly holistic best practice care.

## 48 Investigating the Impact of Patient Lateness on the Podiatry Profession: An International Survey

Thasvhinni Nasendran ^1,^*, Alexis Y. F. Lai^2^, Luke M. Davies^1,3^, and Malia Ho^1^

^1^ *School of Primary and Allied Health, Monash University, Frankston, Victoria, Australia*, ^2^ *Rehabilitation Department, National University Hospital, Singapore*, ^3^ *Department of Health Sciences, Faculty of Medicine, Health and Human Sciences, Macquarie University, Sydney, New South Wales, Australia*

*jtashnas89@gmail.com

**Background:** Podiatrists are crucial for managing lower limb pathologies, and effective scheduling is vital for allocating adequate consultation time based on patient conditions. While occasional late arrivals might not significantly impact services, frequent lateness can disrupt patient flow and quality of care. This study explored the global impact of patient lateness on podiatry practices, assessed current strategies to manage it, and evaluated their effectiveness.

**Methods:** An international cross‐sectional online survey was conducted between January and March 2024.

**Results:** The survey, which garnered 201 responses from podiatrists worldwide, revealed that over 90% of podiatrists experienced disruptions in their clinic workflow due to late patients. Common reasons for lateness included traffic issues and difficulties with parking. SMS reminders emerged as the most effective tool for reducing tardiness. Over half (59.3%) of podiatrists implemented a 10‐min grace period before rescheduling late appointments, which effectively reduced lateness by 50%. However, some podiatrists refrained from rescheduling to avoid worsening patients' conditions or dealing with complaints. Additionally, many podiatrists reported a lack of managerial support in handling late patients.

**Conclusion:** The frequency of late arrivals in podiatry is similar to other health professions and negatively impacts clinic workflow and staff morale. Enhanced managerial support is needed to better manage late patients, allowing podiatrists to concentrate on their clinical responsibilities.

## 49 Cloud Based Integration of the Foot Health Status Questionnaire (FHSQ) Outcome Measure Into Daily Clinical Practice

Paul Bennett ^1,^*, Eddie Price^2^, Saul Brandt^2^, Ned Benjamin^2^

^1^ *Queensland University of Technology, Brisbane, Queensland, Australia*, ^2^ *eHealthier, Queens Park, New South Wales, Australia*

*p.bennett@qut.edu.au

**Background:** Patient reported outcome measures (PROMs) have been identified by the Australian Commission on Safety and Quality in Healthcare as being important in sensitising patients to health issues, facilitating the tracking of health outcomes over time and enabling comparisons between an individual's outcome and those of other patients with the same health condition [1]. PROMs maybe either generic, capturing a broad range of health domains, or region specific. The thirteen item FHSQ specifically captures objective data about the domains of foot related physical pain, movement capacity based on foot function, footwear related quality of life issues and the individual's general perception of foot health [2]. The FHSQ has been translated into 9 languages and utilised in clinical trials spanning investigation into diabetic foot disease, foot health during pregnancy, efficacy of different orthoses design and, lower limb involvement in multiple sclerosis. Until recently, the FHSQ has been primarily employed in clinical trials, however with advancement in understanding the properties of the FHSQ and cloud based technology, barriers to widespread adoption in routine clinical practice can now be overcome.

**Methods:** The patient/population, intervention, comparison and outcomes (PICO) framework was used on 139 randomised controlled, observational cohort, validation and cross sectional studies providing data on more than 3600 patients [3]. Data was analysed to enable development of international reference ranges and calculation of treatment time trends [4].

**Results:** Presented here is a framework for integrating a secure web‐based IT platform with a time‐honoured PROM of proven utility. Combining the concept of healthy reference ranges adjusted for age and gender with minimally important clinical difference and percentage change in health status over time, a new clinical ‘flux’ index has been developed. Using a series of coloured cues, this approach enables clinicians to quantitatively visualise patients' health and assess impact of treatment in a matter of seconds.

**Conclusions:** Shown here is the integration of 25 years of empirical quality of life research into an easy to use, secure IT platform. This new approach facilitates quick and accurate clinical testing, supports patient communication, deepens clinician understanding of treatment, improves health outcomes and enables significantly improved patient and practitioner confidence.

**References**

1. Australian Commission on Safety and Quality in Health Care, https://www.safetyandquality.gov.au/.
2. P. J. Bennett, C. Patterson, S. Wearing, and T. Baglioni, “Development and Validation of a Questionnaire Designed to Measure Foot‐Health Status,” *Journal of the American Podiatric Medical Association* 88, no. 9 (1998): 419–428, 10.7547/87507315‐88‐9‐419.
3. G. M. Tawfik, K. A. S. Dila, M. Y. F. Mohamed, et al., “A Step by Step Guide for Conducting a Systematic Review and Meta‐Analysis With Simulation Data,” *Tropical Medicine and Health* 47, no. 1 (2019): 46, 10.1186/s41182‐019‐0165‐6.
4. D. T. Holmes and K. A. Buhr, “Widespread Incorrect Implementation of the Hoffmann Method, the Correct Approach, and Modern Alternatives,” *American Journal of Clinical Pathology* 151, no. 3 (2019): 328–336, 10.1093/ajcp/aqy149.

## 50 Female Recreational Runners' Perspectives of the Risk Factors for Running‐Related Injury: A Qualitative Exploration

Emily Authurs^1^, Vivienne Chuter^2^, Benjamin Peterson^1^^,^*

^1^ *Department of Podiatry, School of Health, Medical and Applied Sciences, CQUniversity, Rockhampton, Queensland, Australia*, ^2^ *School of Health Sciences, Western Sydney University, Campbelltown Campus, Sydney, New South Wales, Australia*

*Emily.Authurs@cqumail.com

**Background:** Recreational running is a popular choice of physical activity with many health benefits. However, the incidence of running related injury is high. Previous research has attempted to understand the aetiology of running‐related injury with the most well‐established risk factors being previous injury and training errors. However, previous reductionist approaches to identifying running related injury risk factors have been largely unsuccessful. Injury needs to be better understood in its context to build evidence on the complex phenomenon of running‐related injury, therefore qualitative research is essential.

**Methods:** Eleven female recreational runners (47.6 ± 20.0 years of age, 17.5 ± 12.6 years running experience) participated in one of three focus groups regarding running‐related injury risk, prevention, management, and health seeking behaviours. Focus group data were analysed using Braun and Clarke's six phase reflexive thematic analysis approach.

**Results:** Five interconnected themes emerged: ‘the run’, ‘the runner’, ‘the injury’, ‘the networks’, and ‘the gear’. ‘The run’ explained the specific aspects of individual running sessions which could be potentially injurious. ‘The runner’ described individually identified intrinsic and extrinsic factors which influenced running participation and running‐related injury risk. ‘The injury’ encompassed how a runner may recognise and adapt to running‐related injury. ‘The networks’ identified the knowledge hubs which a runner may source, the connectivism with others, and the use of freely available resources to guide running‐related injury prevention and/or management.

**Conclusions:** The female recreational runners included in this study described using processes of self‐regulation, informed by both past advice and past experience, in order to prevent and manage running‐related injury. It was also identified that injury risk was influenced by intrinsic and extrinsic factors which were not within the runners' control. Future injury prevention programs should be responsive to the perspectives of female recreational runners' and take end‐user co‐design approaches to promote their efficacy. Clinicians working with recreational runners should be mindful of the runners' active role in management of their own injury risk, and should enable runners with appropriate resources and education to make informed training decisions.

## 51 Addressing the Peri‐Wound Intact Skin of Hard‐to‐Heal Diabetic Foot Ulcers With Topical Red Deer Conditioned Media (PTT‐6) Skin Conditioner: A Case Series on Clinical Efficacy

Kimberley Leow ^1,^*, Leon Timothy Charles Alvis^1^, Bradley Edmond Charles Yuen^1^, Leighton Cheng^1^, Shiqi Chiam^1^, Siti Nurfarahdillah Binte Abdul Razak^1^, Gerard Benjamin Evans^1^, Kenneth Koh^1^, Ivor Jiun Lim^2^, Toan Thang Phan^2^, Keng Lin Wong^3^

^1^ *Podiatry Department, Sengkang General Hospital, Singapore Health Services, Singapore*, ^2^ *CellResearch Corporation, Singapore*, ^3^ *Island Orthopaedics, Singapore*

*kimberley.leow.wenxian@skh.com.sg

**Background:** The healing of diabetic foot ulcers (DFUs) can be hindered by the susceptibility of the surrounding intact skin to pro‐inflammatory proteases. A conditioned media, known as PTT‐6, derived from mesenchymal stem cells found in the lining of red deer umbilical cords, has been formulated to protect the intact peri‐wound skin of DFUs. The aim is to evaluate the clinical effectiveness of PTT‐6 in managing peri‐wound intact skin in hard‐to‐heal DFUs.

**Methods:** Patients with DFUs that persisted for over 3 months were divided into two subgroups. The active wound group received standard‐of‐care treatment protocol followed by PTT‐6 application around the peri‐wound area, while the maintenance wound group applied PTT‐6 media over the healed wound site.

**Results:** Forty cases were recorded, of which 22 (55%) were included in the active wound group. The majority were male (75%, *n* = 30) and vast majority had cardiovascular risk factors, including diabetes mellitus (100%, *n* = 40), hyperlipidaemia (82.5%, *n* = 33), and hypertension (77.5%, *n* = 31). Most patients had forefoot wounds (80%, *n* = 32) on the plantar aspect (82.5%, *n* = 33). The patients in the active wound group had chronic DFUs for a mean of 218 ± 201 days. Of those treated with PTT‐6 media, 68.4% (*n* = 13) achieved complete wound healing within a mean duration of 69 ± 50 days. Additionally, most patients in the maintenance wound group remained ulcer‐free at 3 months (91.7%, *n* = 11) and 6 months (66.7%, *n* = 6).

**Conclusions:** The study results suggest that PTT‐6 media may serve as an additional treatment modality for enhancing the microenvironment at the peri‐wound intact skin site. This could indirectly facilitate wound healing by preserving the integrity of the peri‐wound intact skin.

## 52 Consumer Engagement to Improve Foot/Ankle Orthopaedic Waiting‐List Management: What Do Patients Want?

Caitlin Swalwell ^1,^*, Tom Walsh^1^, Paul Butterworth^2^, Sankalp Khanna^3^, Louise Ryall^4^, Jenna Mostyn^5^, Karen Holt^6^, Taylor Wilde^6^, Simon Platt^2^

^1^ *Research Office, Gold Coast Hospital and Health Service, Southport, Queensland, Australia*, ^2^ *Department of Orthopaedics and Trauma, Gold Coast Hospital and Health Service, Southport, Queensland, Australia*, ^3^ *The Australian e‐Health Research Centre, CSIRO Health & Biosecurity, Black Mountain, Australian Capital Territory, Australia*, ^4^ *Transformation Delivery, Gold Coast Hospital and Health Service, Southport, Queensland, Australia*, ^5^ *Divisional Analyst and Reporting Team, Gold Coast Hospital and Health Service, Southport, Queensland, Australia*, ^6^ *Consumer Author, Queensland, Australia*

*caitlin.swalwell@health.qld.gov.au

**Background:** Hospital waiting lists for orthopaedic foot/ankle complaints are extensive and most patients experience long waits for surgical consultations. Allied‐health professionals, like podiatrists, are increasingly being utilised to provide clinical triage for non‐urgent presentations with positive effect; however, many patients still require a surgical opinion, and health services are continuously seeking strategies to improve patient flow. This mixed‐methods study aimed to (i) determine what information is important for patients waiting for a surgical consultation regarding their foot/ankle complaint and (ii) gauge patient perceptions around the use of artificial intelligence and machine learning in the provision of care and the prediction of treatment outcomes.

**Methods:** Two cohorts of patients referred to an orthopaedic foot and ankle clinic at a tertiary‐level health service in Queensland were recruited: Patients on the current orthopaedic waiting‐list, and those who underwent foot/ankle surgery within the last 24‐months. Qualitative assessment consisted of individual semi‐structured interviews to explore patient preferences whilst waiting, and thoughts on using algorithms to inform care decisions. Quantitative assessment involved questionnaires on demographics, foot pain (via the Manchester‐Oxford Foot and Ankle Questionnaire [MOxFQ]), and health‐related quality of life.

**Results:** Eighteen participants (13 women) consented to participate in this study. Four were currently wait listed (all with non‐urgent presentations), and 14 previously underwent foot/ankle surgery (6 with semi‐urgent and 8 with non‐urgent presentations). Ninety‐four percent reported high levels of current foot pain on the MOxFQ. Several themes emerged during interviews. Regarding wait‐list experiences, the major theme identified was communication. Most participants reported a need for clearer, more frequent communication regarding their referral status and estimated time to treatment, and an ability to communicate changes in their condition. Regarding perceptions of artificial intelligence, the major theme identified was knowledge. Most participants wanted to know specifically which data are inputted into any algorithm used to make predictions and support clinical decision‐making.

**Conclusions:** Podiatrists and other health professionals involved in managing patient waitlists or intending to use algorithms to inform patient care or predict outcomes can apply these findings in their own practice by ensuring (i) frequent communication with patients regarding their referrals and (ii) transparency regarding the use of patient data.

## 53 Podiatrists' Preferences for the Content and Delivery of a Pre‐Registration Podiatry Program: A Survey of Podiatry Graduates

Hazel Chua^1^, Antoni Fellas^1,2^, Andrea Coda^1,2^, Fiona Hawke ^1,2,^*

^1^ *Podiatry Department, School of Health Sciences, College of Health, Medicine and Wellbeing, University of Newcastle, Ourimbah, New South Wales, Australia*, ^2^ *Equity in Health and Wellbeing Research Program, Hunter Medical Research Institute, Newcastle, Australia*

*Fiona.Hawke@newcastle.edu.au

**Background:** Understanding podiatrists' perceptions of their undergraduate education is important to ensure that educational content and delivery meets the needs of the current workforce to inform future planning. This study aims to explore podiatrists' perceptions of their undergraduate podiatry training and their preferences regarding educational content and delivery.

**Methods:** We conducted an online survey of podiatry graduates from the University of Newcastle, Australia. Data were analysed using descriptive statistics and Fisher's exact test to compare responses between groups. Qualitative responses were analysed using inductive content analysis.

**Results:** A total of 114 registered podiatrists responded. Nail avulsions, business management and modifying orthoses were perceived as being given insufficient time and focus in undergraduate training, with a higher proportion of private (71%) compared to public (33%) podiatrists reporting business management as lacking (*p* = 0.02). There was strong support for embedding endorsed scheduled medicines training within the program (80%), and for delivering theoretical content face‐to‐face rather than online. Inductive content analysis revealed four areas to be emphasised in future curricula: modern technologies, biomechanics, wound care, and routine podiatric care. Potential strategies to reduce examination stress included mock assessments, changed assessment weighting, reduced exam structure rigidity, and reducing assessor bias.

**Conclusion:** This study provides insights into Australian podiatrists' preferences for pre‐registration curricula. Topics to emphasise in future curricula include greater manual skills and business training, modern technologies, biomechanics and routine podiatric care. Our results suggest exercising caution with over‐reliance on online delivery. These findings provide valuable guidance for future curricula in a context of declining student numbers and increasing healthcare demands.

## 54 Charcot Foot Deformity and Wound Prevention via Objective Data Collection to Aid in Better Footwear and Orthosis Design

David Sutton*

*Bilby shoes and orthosis, Thomastown, Victoria, Australia*

*bilbyshoes@live.com.au

The gold standard for treating Charcot foot is the Total Contact Cast (TCC), which provides offloading, stabilisation, and protection. The TCC design ensures full contact with the skin, distributing pressure evenly across the foot and reducing the risk of trauma or ulceration. Its key feature is placing the foot in a planta‐grade position with no heel pitch or toe spring to reduce stress on the foot's structure and skin.

This presentation explores how to incorporate some of the TCC features into footwear and orthotics, focusing on how we assess individual patients and measure outcomes. Accurate assessment is essential for determining the need for this approach, which can prevent further ulceration and complications related to Charcot deformity. It is logical to apply these same features to custom footwear.

We use 3D laser scan data and barefoot pressure analysis to objectively measure the shape, function, and pressure distribution of a patient's feet. These technologies enable personalised footwear design tailored to the patient's unique foot biomechanics and pressure needs. After designing the custom orthosis, we conduct a trial fitting to ensure proper alignment and support. In‐shoe pressure mapping during the gait cycle helps assess foot interaction with the footwear and identifies areas of excessive pressure. Adjustments are made to redistribute pressure and improve symmetry, aiming for peak pressure below 200 kPa.

We designed a lace‐up ankle boot using 3D laser scans and CAD/CAM technology. The boot had no heel pitch or toe spring, similar to a TCC. A vacuum form fitting ensured the correct fit before finalising the footwear. The orthosis was made from various materials based on the patient's foot. Feedback was collected throughout the process, and the patient was educated about each step.

The final footwear resulted in peak pressure below 200 kPa, wound healing, and improved patient adherence. The patient reported better balance, stride, and confidence. Ongoing reviews will be conducted per DFA guidelines.

## 55 Plantar Heel Pain Is Not Associated With Fatty Infiltration of the Abductor Digiti Minimi Muscle on MRI: A Cross‐Sectional Observational Study

John Chen ^1,^*, John Osborne^2^, Hylton Menz^2^, Mandy Abbott^1^, Karl Landorf^2^

^1^ *School of Health and Life Sciences, Glasgow Caledonian University, Cowcaddens Road, Glasgow, UK*, ^2^ *Discipline of Podiatry, School of Allied Health, Human Services and Sport, La Trobe University, Victoria, Australia*

*john26chen@gmail.com

**Background:** It has been proposed that fatty infiltration—or fatty atrophy—of the abductor digiti minimi (ADM) muscle of the foot is associated with entrapment of the first branch of the lateral plantar nerve (i.e., Baxter's neuropathy) as part of plantar heel pain (PHP), however this association has not been rigorously investigated. The aim of this study was to determine if there is an association between PHP and fatty infiltration of the ADM.

**Methods:** This cross‐sectional observational study compared 50 participants with PHP (PHP group) to 25 participants without PHP (no‐PHP or control group) who were matched at recruitment for age (± 5 years), sex and body mass index (BMI) (± 10%). Fatty infiltration of ADM was assessed on all participants using magnetic resonance imaging (MRI). Prior to assessment, four grading scales of fatty infiltration were investigated for reliability by two independent assessors and the most reliable scale was chosen for the primary data analysis. Following this, participant characteristics were compared between the PHP group and the no‐PHP group to ensure there were no significant differences between the two groups in characteristics that could have confounded the findings. The association between PHP and fatty infiltration was analysed using the Chi‐square test.

**Results:** The four‐point grading scale proposed by Recht et al. was found to be the most reliable scale. There were no significant differences (*p* > 0.05) for important participant characteristics (e.g., age, sex and BMI) between the two groups that could have confounded the findings. No significant association was found (*χ* ^2^ = 6.176, df = 3, *p* = 0.103) between PHP and fatty infiltration according to the four‐point Recht et al. grading scale.

**Conclusion:** After accounting for age, sex and BMI, there was no association between PHP and fatty infiltration of ADM. Therefore, fatty infiltration of ADM is likely to be an incidental finding on MRI rather than a diagnostic sign of PHP, where Baxter's neuropathy may be present. Accordingly, clinicians should not focus on fatty infiltration of ADM on MRI to diagnose Baxter's neuropathy, or view it as a surrogate marker, particularly when surgery for PHP is being considered.

## 56 Retail and Therapeutic Footwear Use in People With Diabetes Mellitus: A Systematic Review

Zainab Al Modhefer ^1,2,^*, Sean Lanting^1,2^, David Simmons^3^, Vivienne Chuter^1,2^

^1^ *School of Health Sciences, Western Sydney University, Campbelltown, New South Wales, Australia*, ^2^ *School of Health Sciences, University of Newcastle, Callaghan, New South Wales, Australia*, ^3^ *School of Medicine, Western Sydney University, Campbelltown, New South Wales, Australia*

*z.al-modhefer@westernsydney.edu.au

**Background:** Footwear has been established as both a key factor in the development of diabetes‐related foot ulcer (DFU) and an effective therapeutic intervention for their prevention. Poorly fitting footwear can result in foot trauma leading to DFU development. However well‐fitting retail footwear and therapeutic footwear are recommended to reduce risk of DFUs. This systematic review was undertaken to establish factors influencing choice of retail footwear, as well as the factors of adherence and non‐adherence to therapeutic in people with diabetes.

**Methods:** Medline, EBSCO Megafile Ultimate and Cochrane library databases were searched until August 2024 to identify studies investigating factors influencing choice of retail footwear in people with diabetes. A second search was undertaken using the same databases to identify studies investigating factors affecting adherence and non‐adherence to therapeutic footwear in adults with diabetes. Studies were deemed ineligible if they only reported demographic correlations with footwear choice or were conducted in populations without diabetes. Methodological quality of eligible articles was assessed using the *Mixed Methods Appraisal Tool* (*MMAT*).

**Results:** One article was retrieved investigating factors influencing retail footwear choices in people with diabetes. The main factors affecting footwear choice were availability and affordability. Seventeen articles were identified investigating factors affecting adherence and non‐adherence to therapeutic footwear in people with diabetes. Appearance of footwear was the most frequently reported variable to result in non‐adherence, followed by shoe weight, and the shoe deemed inappropriate for social use. Increased footwear adherence was associated with self‐perceived poor foot health, and fear of risk of ulceration/amputation and deformities.

**Conclusions:** This review identified availability and affordability as the main factors affecting choice of retail footwear by people with diabetes. More research in this area is required to determine factors influencing retail footwear choice in people with diabetes. The factors of adherence and non‐adherence, such as excessive weight of the shoe, poor appearance and suitability for social use, should be considered in the design, manufacture, and prescription of therapeutic footwear to improve rates of wear compliance.

## 57 How Podiatrists Navigate Diagnostic Uncertainty in Children's Chronic Lower Limb Pain. Learning From Each Other

Jessica Coventry^1,^*, James J. Welch^2^, Verity Pacey^1,3^, Binh Ta^1^, Elizabeth Sturgiss^1^, Mitchell Smith^1^, Cylie M. Williams^1^

^1^ *Monash University, Frankston, Australia*, ^2^ *Ablefeet Ltd, Surrey, UK*, ^3^ *Macquarie University, Macquarie Park, Australia*

*jess.coventry@monash.edu

**Background:** Chronic lower limb pain is common in children and adolescents and is frequently managed by podiatrists. There is limited research with podiatrists and how they manage chronic lower limb pain in children, especially in the presence of diagnostic uncertainty. This study aimed to explore the management strategies including language that podiatrists report using to address the pain experience of children with chronic lower limb pain. The secondary aim was to investigate if and how the reported management strategies used by podiatrists to address the pain varies based upon the level of diagnostic uncertainty.

**Methods:** We recruited podiatrists during the Australian Podiatry Conference, 2023 during a workshop. All those attending the workshop were eligible to participate. Podiatrists were presented with three vignettes where all stories described a child's journey and impact of chronic pain present in the lower limb (Child 1 presented with calcaneal apophysitis, Child 2, with Juvanile Idiopathic Arthritis, and Child 3, generalised primary musculoskeletal pain. Podiatrists were then asked to discuss their certainty in the child's diagnosis and any approaches to explaining and managing the child's pain. Feedback was provided during the workshop on responses from other tables with a real time graphical recorder. Subsequently, the audio files were transcribed and analysed using thematic analysis.

**Results:** There were 48 podiatrists join eight focus groups over 90 min, with written consent for audio recording group discussion. Three key themes were generated for how podiatrists proposed managing chronic pain in the presence of diagnostic uncertainty: Language strategies, non‐verbal communication strategies and treatment strategies. Podiatrists used similar language strategies across all three vignettes and supported their language strategies with non‐verbal communication strategies. Podiatrists also discussed activity modification, passive and self‐care strategies and building a team as the treatment strategies they would use. Where there was increasing diagnostic uncertainty, podiatrists expressed a willingness to continue seeking a diagnosis.

**Conclusions:** Not all clinical presentations associated with chronic pain have a clear diagnosis. This research highlights how diagnostic uncertainty can impact communication and management approaches. This provides an opportunity to rethink how to approach clinical consultations when children present with chronic pain.

## 58 The Walk Strong, Walk Tall Program: Preventing Diabetes‐Related Foot Complications for Our Mob in South Australia

Courtney Hammond^1,^*, Saraid Martin^1^, Natalie Morgan^1,2^

^1^ *Wardliparingga Aboriginal Health Equity, SAHMRI, Adelaide, South Australia, Australia*

*Courtney.Hammond@sahmri.com

**Background:** Aboriginal and Torres Strait Islander people continue to be disproportionately affected by diabetes and diabetes‐related foot complications (DRFC). The Walk Strong, Walk Tall program is multi‐faceted in preventing DRFC by strengthening workforce numbers, cultural capability and professional skills and knowledge, increasing education opportunities around the importance of foot health, improving access to and culturally appropriateness of podiatry services across South Australia (SA).

**Methods:** The program aims to address multiple contributing factors for DRFC for Aboriginal and Torres Strait Islander people in SA. The community arm of the program focuses on strengthening community knowledge of DRFC, the importance of preventative care practises and podiatry career pathways. The workforce arm builds capacity of primary health service staff around clinical foot screening, promoting podiatry career pathways and increasing overall knowledge of DRFC. Finally, the system arm works to improve the health system more broadly in supporting Aboriginal and Torres Strait Islander people within the primary, secondary and tertiary health systems.

**Results:** Through community education events, school visits and the development of My Feet children's book, the program has been providing education around DRFC across South Australia. Walk Strong, Walk Tall have been providing clinical and cultural responsiveness training, as well as doing education sessions within non‐clinical workplaces. The program has improved access to podiatry services in rural and regional locations and is in the process of developing amputation resources which have been co‐designed with Aboriginal lived experience amputees.

**Conclusions:** Prevention is integral when working to improve health outcomes for Aboriginal and Torres Strait Islander people. Through strengthening community knowledge around the importance of foot health and advocacy efforts, building capacity of clinical workforce and advocating for a stronger, more culturally appropriate health system, we can prevent DRFC for our mob.

## 59 The F‐Words Relating Symptomatic Flexible Flat Feet. A Scoping Review

Jovana Urukalo ^1,^*, Helen Banwell^1^, Cylie Williams^2^, Stewart C. Morrison^3^, Saravana Kumar^1^

^1^ *Allied Health and Human Performance, University of South Australia, Adelaide, Australia*, ^2^ *Monash University, School of Primary and Allied Health, Frankstown, Victoria, Australia*, ^3^ *Department of Population Health Science, School of Life Course and Population Sciences, Faculty of Life Sciences and Medicine, King's College London, London, UK*

*Jovana.urukalo@unisa.edu.au

**Background:** Flexible flat feet are one of the most common musculoskeletal concerns presenting to paediatric health services, despite this being an expected finding in children under 10 years and only requiring management when symptoms are associated. Understanding which symptoms are associated with symptomatic presentations of flexible flat foot in children will provide clarity in identifying those that require further assessment and/or intervention.

**Methods:** A scoping review of the literature was conducted to gather all reported symptoms related to symptomatic flexible flat foot in the child. Data was mapped using the ‘F‐words’ framework, a child friendly, six‐item tool (*fitness, functioning, friends, family, fun* and *future*), based on the International Classification of Functioning, Disability and Health Framework 11 (ICF‐11).

**Results:** From the 133 articles included, 42 broad categories of symptoms were identified, which were allocated into five of the six ‘F‐words’ categories. Of these, pain was the most reported symptom, followed by symptoms associated with reduced lower limb function (altered gait patterns, reduced balance and stability and increased tripping), fatigue and reduced participation. Other less frequently reported symptoms include callus formation, night pain and cramps. When present, these symptoms may occur independently or may co‐exist at the same time.

**Conclusions:** A multitude of symptoms are reportedly associated with symptomatic flexible flatfoot in the child, with no discernible pattern or coherence noted. Further research should examine development and progression of symptoms and seek to better understand causality of relationship between symptoms and foot posture.

## 60 The Impact of the ‘Learning‐Bytes’ Program on Perceptions and Confidence of Allied Health Clinicians Involved in Student Clinical Placement Supervision

Helen Banwell ^1,^*, Anna Phillips^1^, Sarah McMullen‐Roach^1^, Emma Hiscock^2^, Kitty Pham^3^, Gisela van Kessel^4^, Caroline Fryer^1^

^1^ *Allied Health and Human Performance Unit, University of South Australia, Adelaide, South Australia, Australia*, ^2^ *Department of Rural Health, University of South Australia, Adelaide, South Australia, Australia*, ^3^ *Clinical and Health Sciences Unit, University of South Australia, Adelaide, South Australia, Australia*, ^4^ *UniSA Online, University of South Australia, Adelaide, South Australia, Australia*

*helen.banwell@unisa.edu.au

**Background:** The quality of clinical placement is known to impact students' work readiness; however, clinicians often report a lack of confidence and support in creating effective learning environments. The ‘Learning‐bytes’ program, co‐designed with an expert reference group, is a flexible, online resource, developed for allied health clinicians supervising students. Comprising five stand‐alone ‘bytes’, each taking approximately 45 min to complete, this program covers topics such as *feedback that gives back* and *when things go wrong.* Each byte contains a podcast with content experts, video and written resources, and a short quiz. Participants completing all five quizzes attain certification. This study investigated how engagement with the program impacted clinicians' perceptions and confidence.

**Methods:** A mixed‐method study collected measures of interest via three surveys, and a focus group, over 15 weeks. Evaluations explored usability (ease of use and ability to meet learning needs) and change in participant knowledge, confidence, perception of best practice, and willingness to take students. Quantitative data are displayed as descriptive statistics with a two‐tailed *t*‐test applied to comparisons. Qualitative data themes were identified via summative content analysis.

**Results:** Of 123 participants enrolled during the study period (77% female, 70% aged 40 years or younger), 64 completed the program and obtained certification. Forty‐nine participants responded to the usability survey, where over 90% strongly agreed or agreed that the program was easy to navigate (*n* = 45) and met their learning needs (*n* = 46). Five‐point Likert scale pre and post‐survey comparisons (*n* = 32) identified statistically significant improvements in knowledge (3.31 [0.82] vs. 4.13 [0.34] *p* = 0.00), confidence (3.59 [0.71 vs. 4.09 [0.53] *p* = 0.0) and perception of best practice (3.59 [0.76] vs. 4.09 [0.44] *p* = 0.00). No change was noted in participants' willingness to take students (4.41 [0.71] vs. 4.59 [0.56] *p* = 0.14). Focus group outcomes identified attributes, practices, and impact as primary themes associated with quality clinical education.

**Conclusions:** From the clinician's perspective, engagement in the Learning‐bytes program was considered easy and impactful and resulted in improved student supervision‐related skills. The existing high willingness to take students remained unchanged.

## 61 Optimal Temperature Stabilisation Period After Total Contact Cast (TCC) Removal for Assessing Dermal Temperatures in Active Charcot Neuro‐Osteoarthropathy

Justin Bradley ^1,^*, Mollie Rumble^2^, Jennifer Wong^3^, Ming Yii^4^, Michelle R. Kaminski^6,7,8^

^1^ *Department of Podiatry, St Vincent's Hospital Melbourne, Melbourne, Victoria, Australia*, ^2^ *Department of City Futures, City of Stonnington Council, Malvern, Victoria, Australia*, ^3^ *Diabetes & Vascular Medicine Unit, Monash Health, Melbourne, Victoria, Australia*, ^4^ *Department of Vascular and Transplant Surgery, Monash Health, Melbourne, Victoria, Australia*, ^5^ *Department of Surgery, School of Clinical Sciences at Monash Health, Monash University, Melbourne, Victoria, Australia*, ^6^ *Department of Podiatry, Monash Health, Melbourne, Victoria, Australia*, ^7^ *School of Primary and Allied Health Care, Monash University, Melbourne, Victoria, Australia*, ^8^ *Discipline of Podiatry, School of Allied Health, Human Services and Sport, La Trobe University, Melbourne, Victoria, Australia*

*Justin.bradley@svha.org.au

**Background:** Dermal temperature differentials between limbs are used to monitor disease progression and guide safe removal of immobilisation in Charcot neuro‐osteoarthropathy (CNO). Despite the wide clinical use of dermal thermometry, there is a lack of evidence on the optimal temperature stabilisation period after removal of immobilisation devices, such as total contact casts (TCC). This study aimed to investigate the optimal temperature stabilisation period post removal of TCC for assessing dermal temperatures in active CNO.

**Methods:** Over a 2‐year period, this within‐session repeated measures study recruited 12 adults with active CNO treated with a TCC from a metropolitan high‐risk foot service. Participants were excluded if they had bilateral CNO, an active foot ulcer, major lower limb amputation, peripheral artery disease, or another inflammatory foot condition (e.g., gout, osteomyelitis). In a temperature‐controlled room, dermal temperatures were recorded using an infrared thermometer (Exergen DermaTempTM) after removal of the TCC and footwear. Temperatures were recorded at 10‐min intervals from baseline to 90 min at 10 anatomical locations on each foot. Paired Samples *T*‐tests or Wilcoxon Signed‐Rank Tests were used to explore stabilisation of dermal temperatures at each site across the 10 time‐points.

**Results:** Mean age was 55.1 (SD, 8.9) years, 75.0% were male, and the majority had type 2 diabetes (83.3%). All participants had peripheral neuropathy, and a large proportion had history of foot ulceration (75.0%). The average duration of CNO was 2.9 (SD, 1.7) months, with the left foot (66.7%) more commonly affected. The majority of participants had modified Eichenholtz stage 1 Charcot foot (91.7%) affecting the tarsometatarsal joints (58.3%) and midtarsal joints (83.3%). Trauma was a common Charcot precipitant (41.7%), although this was often unknown (58.3%). Overall, dermal temperatures had stabilised at 40 min for both feet.

**Conclusions:** This is the first study to explore the optimal temperature stabilisation period for assessing dermal temperatures in active CNO. Testing dermal temperatures after 40 min appears to be an appropriate resting time for most locations to reach thermal equilibrium. While this approach may improve the accuracy of dermal thermometry, the time period may not always be feasible in clinical practice.

## 62 Comparing the Foot Disease Burden and Its Impact on Daily Living Between Rheumatoid Arthritis, Axial Spondyloarthritis, and Psoriatic Arthritis in Singapore: A Cross‐Sectional Study

Eunice Yang^1^^,^*, Deborah Turner ^1,^*, Kate Carter^2^, Peter Cheung^3,4^, Manjari Lahiri^3,4^

^1^ *Podiatry, Faculty of Health, School of Clinical Sciences, University of Technology, Brisbane, Queensland, Australia*, ^2^ *Department of Podiatric Medicine and Surgery, School of Allied Health, The University of Western Australia, Crawley, Western Australia, Australia*, ^3^ *Division of Rheumatology, Department of Medicine, National University Hospital Singapore, Singapore*, ^4^ *Department of Medicine, Yong Loo Lin School of Medicine, National University of Singapore, Singapore*

*deborah.turner@qut.edu.au

**Background:** Foot pathologies are highly prevalent in people with inflammatory arthritis. Despite this similarity, rheumatoid arthritis, axial spondyloarthritis, and psoriatic arthritis present distinct manifestations within the foot, which may result in disease‐specific foot disease burdens and varying functional and psychosocial limitations. Currently, research aimed at understanding the differences in the impact of foot involvement in inflammatory arthritis is lacking, especially within Asian populations. This study aims to compare the foot‐related disease burden and its impact on activities of daily living among people diagnosed with rheumatoid arthritis, axial spondylarthritis, and psoriatic arthritis in Singapore.

**Methods:** This study is a retrospective secondary analysis of cross‐sectional data collected from the One‐Stop Arthritis Clinic at the National University Hospital in Singapore, between 2016 and 2022. Forty adults from each condition were selected from the database based on age and gender matching. Foot‐ and ankle‐specific characteristics that measured foot disease burden, physical function, and health‐related quality‐of‐life were analysed.

**Results:** Foot‐related disease burden is highly prevalent in all three disease groups, with 45% of all participants experiencing foot problems. A significantly higher proportion of people with psoriatic arthritis and rheumatoid arthritis reported forefoot pain and presented with forefoot structural deformity, compared to people with axial spondyloarthritis (*p*_pain_ = 0.021; *p*_deformity_ = 0.019). People with psoriatic arthritis exhibited significantly higher localised swollen and tender joint counts in the foot compared with the other two conditions (*p* < 0.05). Despite similar levels of physical impairment across groups, a significantly highest proportion of people with psoriatic arthritis reported cessation of weight‐bearing leisure activities (*p* = 0.019).

**Conclusions:** This study identified disease‐specific variations and factors contributing to foot‐related disease burden in inflammatory arthritis subtypes. While the three subtypes show similar global disease activity levels, our findings revealed that people with psoriatic arthritis experience the greatest localised inflammatory burden and functional impairment in the foot. This highlights the urgency to develop foot‐specific outcome measures and tailored management strategies for psoriatic arthritis to prevent the under‐recognition of active disease and optimise patient outcomes.

## 63 Comparison of a Hand‐Held Tablet to a High‐End Console Ultrasound for Identifying Osteoarthritis of the First Metatarsophalangeal Joint

Kate O. Atkinson, Janae M. Barnes, Emily S. Lennon, Ashley Y. W. Leong, Renee N. Silvester*

*School of Allied Health, The University of Western Australia, Crawley, Western Australia, Australia*

*renee.silvester@uwa.edu.au

**Background:** Osteoarthritis (OA) in the first metatarsophalangeal joint (MTPJ) is a common degenerative condition causing joint pain and damage. Ultrasound (US) can be used as a point‐of‐care tool to diagnose OA, however, the high cost and portability of high‐end console (HEC) US devices limit this. Hand‐held tablet (HHT) US devices are cheaper and more portable. Therefore, the primary aim of this study was to assess the performance of a HHT compared with a HEC for detecting osteophytes and synovitis in patients with radiographic or clinical evidence of first MTPJ OA. A secondary aim was to assess inter‐rater reliability between the two US devices. A tertiary aim was to compare perceived image quality of a HHT and HEC device.

**Methods:** Individuals with radiographic (*n* = 16) and clinical (*n* = 10) evidence of the first MTPJ OA underwent ultrasound assessment of the first MTPJ by four examiners, who identified the presence or absence of osteophytes and synovitis based on the validated OA US tool on both HHT and HEC. Inter‐rater reliability between examiners was assessed and grey‐scale static images were then rated by four examiners based on their resolution, detail and image quality on a 10‐point scale.

**Results:** A significant difference between the HHT and HEC for identifying osteophytes within participants was observed (*p* < 0.01). However, there was no significant difference between the two devices for identifying synovitis (*p* = 0.881). A fair to moderate and slight to moderate level of agreement among examiners was demonstrated in the identification of osteophytes and synovitis respectively (ICC 0.33–0.42 and 0.12–0.51 respectively). The resolution, detail, and image quality of HEC was favoured over the HHT with mean differences of 1.7 (95% CI = [1.53–1.66], *p* < 0.001), 1.61 (95% CI = 1.46–1.75], *p* < 0.001), and 1.52 (95% CI = [1.23–1.69], *p* < 0.001) respectively.

**Conclusion:** These findings suggest that the HHT is effective in detecting signs of OA, however, practitioner confidence and its reduced perceived image quality may impact device adoption.

## 64 Intrinsic Foot Muscle Exercise on Improving Symptomatic Early‐Stage First Metatarsophalangeal Joint Osteoarthritis in Western Australians: A Mixed Methods Design

Renee N. Silvester*, Teba Al Taey, Karishma Velugula, Mathew Millar, John Bacus

*Podiatric Medicine and Surgery Discipline, The University of Western Australia, Crawley, Western Australia, Australia*

*renee.silvester@uwa.edu.au

**Background:** This mixed‐methods study investigated the effectiveness of a 4‐week intrinsic foot muscle exercise program for early‐stage first metatarsophalangeal joint (MPJ) osteoarthritis (OA) in reducing pain, improving strength, and range of motion (RoM). It also explored participants' perceptions of exercise as a conservative management technique. A secondary aim was to assess the impact of pain reduction and functional improvement on quality of life (QoL).

**Methods:** Thirteen participants aged 18 and over with radiographically or clinically diagnosed early‐stage first MPJ OA were recruited from the UWA Podiatry Clinic in Perth, Western Australia. Participants performed daily exercises—toe swapping, toe splaying, and short foot exercises—progressing from semi‐weightbearing to full‐weightbearing over 4 weeks. Pain was measured using the Numeric Pain Rating Scale (NPRS), strength with a handheld dynamometer (HHD), RoM with a universal goniometer, and QoL with the Manchester Foot Pain and Disability Index (MFPDI). Measurements were taken at baseline (T0) and post‐intervention (T4). Semi‐structured interviews at T4 explored participants' perceptions of OA and the exercise program. Paired *t*‐tests and Wilcoxon signed‐rank tests analysed the data. Quantitative and qualitative findings were integrated using a sequential explanatory design.

**Results:** Quantitative analysis showed significant pain reduction and dorsiflexion strength improvement. Mean pain reductions were 1.44 for current pain (*p* = 0.004) and 1.75 for average pain (*p* = 0.027). Dorsiflexion strength increased significantly (mean difference: 5.543, *p* = 0.020, Cohen's *d* = 0.836), with no significant changes in plantar flexion strength or RoM. Qualitative findings from nine interviews highlighted improved mobility and strength. Six participants preferred exercise for OA management, while three favoured orthotics, footwear, or medication. All participants continued exercising after the study, citing reduced pain and improved function.

**Conclusions:** The 4‐week intrinsic foot muscle exercise program significantly reduced pain and improved dorsiflexion strength and mobility in early‐stage first MPJ OA. Participants found the program beneficial for pain management and function. The study supports integrating low‐cost, conservative exercise interventions for first MPJ OA management.

## 65 Comparison of Gait and Balance Between Paediatric Survivors of Posterior Fossa Brain Tumours and Typically Developing Children

Matthew Rixon^1^, Stewart Trost^2^, Glen Lichtwark^1^, Gabriel Siqueira Trajano^1^

^1^ *Queensland University of Technology, Brisbane, Queensland, Australia*, ^2^ *University of Queensland, Brisbane, Queensland, Australia*

**Background:** Central nervous system (CNS) tumours are the most common paediatric presentation of solid tumours, representing approximately 20% of paediatric cancers with posterior fossa brain tumours (PFBT) making up 50% of CNS tumours. Recent studies have shown a marked increase in survivorship in paediatric PFBT, however cancer treatment or the original lesion often leave ongoing physical symptoms such as cardiopulmonary disease, renal disease and diminished musculoskeletal sequelae such as muscle weakness and ataxia (5). These physical impairments along with neurocognitive symptoms can diminish participation in social and physical activities, resulting in poorer health outcomes moving into adulthood. Currently much of the evidence has been centred around adulthood quality of life, with a paucity of evidence surrounding the paediatric cohort. The aim of this project is to determine if there are differences in the gait and balance of paediatric PFBTS compared to typically developing controls, as these basic movement patterns required for activities of everyday life are largely unexplored in the paediatric PFBT cohort. Furthermore, we will investigate whether potential differences in gait and balance are related to the age of patients at diagnosis of PFBT. This research will help inform targeted rehabilitation programs to reduce the burden of the disease and treatment to improve quality of life for survivors in adulthood.

**Methods:** This study used a cross‐sectional matched pair design. Participants included 21 paediatric survivors of posterior fossa brain tumours, aged between 5 and 16 years. The control group consisted of typically developing children between the ages of 5–18, age (± 2 years), sex and height matched. Temporal spatial parameter of gait was measured using the GAITRite instrumented walkway, and balance recorded using a Pasco force plate, under six different conditions (Bilateral, unilateral both legs, then repeated on a foam block to create a semi‐dynamic environment.)

**Results:** Data collection has been completed for this project, with data analysis over the next month (Jan–Feb 2025). Statistical models will be used to compare group differences, accounting for matched controls and condition‐specific variability. Based on previous research, It is hypothesised Paediatric PFBTS will have a slower walking velocity, shorter stride length and wider base of gait than the healthy controls and show more signs of ataxia. Furthermore, Paediatric PFBTS will have poorer balance during quiet standing than healthy controls as defined by a longer COP pathway, greater acceleration of the COP and greater area of 95% ellipse.

**Conclusions:** This project seeks to quantify the magnitude of gait and balance impairments in paediatric PFBT survivors. Findings form this study will inform targeted rehabilitation programs or therapeutic interventions (footwear, orthoses or bracing) aimed at improving motor function, increasing stability and enhancing quality of life moving into adulthood. Furthermore, additional studies may expand the knowledge in this area, focused on the kinematic and kinetic gait analysis to further aid in targeted assistance for survivors of posterior fossa brain tumours.

## 66 The Effects of Wearing Textured Versus Smooth Insoles for 4‐Weeks in People With Diabetic Peripheral Neuropathy: A Randomised Controlled Trial

Anna L. Hatton ^1,^*, Mark D. Chatfield^2^, Elise M. Gane^1^, Jayishni N. Maharaj^3^, Thomas Cattagni^4^, Joshua Burns^5^, Joanne Paton^6^, Keith Rome^7^, Graham Kerr^8^

^1^ *School of Health and Rehabilitation Sciences, The University of Queensland, Brisbane, Australia*, ^2^ *Centre for Health Sciences Research, The University of Queensland, Brisbane, Australia*, ^3^ *School of Allied Health Sciences, Griffith University, Gold Coast, Australia*, ^4^ *Laboratory Movement, Interactions, Performance EA 4334, University of Nantes, Nantes, France*, ^5^ *University of Sydney School of Health Sciences, Faculty of Medicine and Health & Children's Hospital at Westmead, Sydney, Australia*, ^6^ *School of Health Professions, Faculty of Health, University of Plymouth, Plymouth, UK*, ^7^ *School of Clinical Sciences, Auckland University of Technology, Auckland, New Zealand*, ^8^ *Movement Neuroscience Group, School of Exercise and Nutrition Sciences, Queensland University of Technology, Brisbane, Australia*

*a.hatton1@uq.edu.au

**Background:** Peripheral neuropathy is one of the most common complications of type 2 diabetes, which can lead to poor balance, walking instability, and falls. Innovative footwear devices designed to stimulate sensory receptors at the feet, could offer a new route to improve motor impairments in people with diabetic peripheral neuropathy. Textured insoles comprising raised nodules designed to augment plantar sensory input, have been shown to enhance balance and walking in ageing and neurological disease populations. The aim of this randomised controlled trial was to determine whether short‐term wear of textured insoles alters balance, gait, foot sensation, physical activity, or patient‐reported outcomes, in people with diabetic peripheral neuropathy.

**Methods:** Fifty‐three ambulant men and women with peripheral neuropathy, secondary to type 2 diabetes, were randomised to wear textured (intervention) or smooth (control) insoles for 4‐weeks. At baseline and post‐intervention, assessments of standing balance (foam and firm surface; eyes open and closed) and level‐ground walking were completed whilst barefoot, wearing shoes only, and two different insoles (textured and smooth insoles). The primary outcome was centre of pressure total sway velocity. Secondary outcomes included other centre of pressure measures, spatiotemporal gait measures, foot sensation, physical activity, and patient‐reported outcomes (foot health, falls efficacy).

**Results:** Wearing textured insoles for 4‐weeks led to improvements in centre of pressure measures when standing on a foam surface with eyes open, relative to smooth insoles (*p* ≤ 0.04). At post‐intervention, the textured insole group demonstrated a 5% reduction in total sway velocity, indicative of greater balance control. The textured insole group also showed a 9‐point improvement in self‐perceived vigour (*p* = 0.03). Adjustments for multiple comparisons were not applied.

**Conclusions:** This study provides some statistical evidence in favour of textured insoles. Wearing textured insoles may alter measures of standing balance, that are suggestive of greater stability, in people with diabetic peripheral neuropathy. Specifically, short‐term wear of textured insoles can lead to improvements in centre of pressure sway measures when standing on a compliant supporting surface. Plantar stimulation, through wearing textured insoles, may also have the capacity to modulate the perception of foot pain, leading to enhanced well‐being in people with diabetic peripheral neuropathy.

## 67 Supporting First Nations Peoples Rights to be Able to Access Appropriate Footwear

Frances B. Elcoate ^1,^*, Danielle Pollock^2^, Greg Fyfe^3^

^1^ *Aboriginal Medical Services Alliance Northern Territory (AMSANT), Darwin, Australia*, ^2^ *Miwatj Health Aboriginal Corporation, Nhulunbuy, Northern Territory, Australia*

*Contracted outreach Podiatrist

*frances.elcoate@amsant.org.au

**Background:** Appropriate footwear is essential for health and well‐being, supporting participation in education, employment and sports, and other physical activities and preventing injuries caused by walking barefoot. People with diabetes, especially in remote NT communities, require protective footwear to prevent injury and serious complications which can lead to amputations. Aboriginal and Torres Strait Islander communities face a 3‐ to 6‐fold increased likelihood of experiencing diabetes‐related foot complications; and the highest rates of diabetes related amputations with a 38‐fold increased risk compared to the wider Australian population. Lack of appropriate footwear is a significant contributor to diabetes‐related foot complications. Appropriate and medical footwear needs to be identified as a critical aid/assistive technology that all people should be able to access, regardless of their location. The South Australian Health and Medical Research Institute (SAHMRI) led Aboriginal and Torres Strait Islander Foot Complications Program was established to improve foot health and reduce amputation rates for Aboriginal and Torres Strait Islander people with diabetes in South Australia, the Top End of the NT and Central Australia, the Kimberley region in Western Australia and Far North Queensland.

**Methods:** Baseline research and stakeholder consultation throughout the NT established a critical need to improve access to affordable footwear. Virtual roundtable discussions and broad community and stakeholder consultation and forums were held to discuss and define enablers and barriers to access to appropriate footwear and create educational resources. AMSANT amplified advocacy through Healthy Living NT and the Australian Podiatry Association to support AMSANT recommendations submitted to the NT Government in 2021 to provide a footwear subsidy. Further consultation, research and feedback across service providers has identified barriers to access and use of appropriate footwear.

**Conclusions:** Appropriate off‐the‐shelf as well as medical grade footwear has been highlighted as a key primary prevention strategy. Challenges in accessing appropriate footwear are cultural, economic and organisational. Cross‐sector partnership and a holistic approach is required to improve access to high quality and affordable appropriate footwear for those who need them. Critical future initiatives include further stakeholder consultation to establish community priorities and further inform strategy and to establish innovative, community led models to improve access to footwear in remote communities.

## 68 Back to Basics: Inclusive Language in Podiatry

Benjamin Bullen^1,^*, Emily Haworth^2^, Lauren Connell^1^

^1^ *University of Galway, Galway, Ireland*, ^2^ *NHS Wales*

*Benjamin.bullen@universityofgalway.ie

**ABSTRACT**

This narrative explores the concept of inclusive language, how to adopt it in clinical practice and what we can do to adapt our communication for the individual presenting for podiatric care. The demand for this article was highlighted at a recent associated webinar, involving the authors (Haworth, Bullen and Connell, 2024). Inclusive language has the potential to impact patient outcomes by reducing communication barriers in the patient‐practitioner relationship. Adopting an inclusive approach allows individuals to be placed firmly at the centre of their health management. As podiatrists, we must demonstrate an awareness of the language we use and how it can impact individuals accessing podiatric care. When we adopt an inclusive approach to patient care, individuals feel heard, accounted for and own their healthcare.

## 69 Tools Used to Assess Wellbeing in People Living With Chronic Lower Extremity Wounds: A Scoping Review

Lauren Connell*, Benjamin Bullen, Ellen Kirwan, Claire MacGilchrist, Caroline McIntosh

*Department of Podiatric Medicine, University of Galway, Galway, Ireland*

*l.connell3@universityofgalway.ie

**Background:** Optimising wellbeing for those living with chronic wounds is essential to achieve holistic, patient‐centred care and improve patient outcomes. However, despite a plethora of outcome measure tools to assess domains of wellbeing for individuals living with chronic wounds, there is lack of standardisation across these tools, and the consideration of wellbeing as a multidimensional concept is often lacking.

The objectives of this scoping review were to:

- identify patient‐reported outcome measures used to assess wellbeing in people living with chronic lower extremity wounds, diabetic foot ulceration (DFU) and venous leg ulceration (VLU),
- evaluate the subdomains of wellbeing (physical, psychological, social and spiritual) assessed within existing tools.

**Methods:** A scoping review was conducted drawing on methods defined by the Joanna Briggs Institute, and in accordance with the Preferred Reporting Items for Systematic Reviews and Meta‐Analyses Extension for Scoping Reviews (PRISMA‐ScR). Eligibility included studies, of any methodology reporting primary data on wellbeing in adult participants living with DFU and VLU.

**Results:** One hundred and forty‐five articles were included in this review; 83 studies focused on diabetes‐related foot ulceration (DFU), with 52 studies focused on venous leg ulcers (VLU). Across all studies, a range of tools were identified to assess quality of life and domains of wellbeing. Quality of life and physical parameters of wellbeing dominated the areas of measurement. A minority of tools assessed all four domains of wellbeing.

**Conclusions:** Due to a plethora of outcome measure tools, assessment of wellbeing is variable and inconsistent lacking a standardised approach. Furthermore, there is a lack of multidimensional evaluation of wellbeing in people living with common lower extremity wound with evident gaps in the assessment of the sub‐domains of spiritual and social wellbeing which were frequently overlooked.

## 70 One Small Step for Podiatrists, One Giant Step for Safety: Tapping Into the Potential of Sharps Injury Data

Chamindika Konara, Shreya Singh*

StaffAndPatientSafety.org

*shreya@staffandpatientsafety.org

**ABSTRACT**

‘I am right‐handed, in 2015 I was stabilising a patient's foot with my left hand, and debriding with my right hand. I dropped the scalpel and instinctually reached out to catch it with my left hand. Which I did …. I still have altered sensation on the lateral side of my L/4 finger!’ A seemingly minor scalpel cut, to Molly a practicing podiatrist, which should have been reported, became a lasting injury. Podiatrists, who handle scalpels, face the greatest risk of sharps injuries in healthcare settings. Despite this up to 50% of sharps injuries in Australia go unreported. Worse still is the staggering injury rates amongst podiatry students. A 2024 study, of pre‐registered nursing, medicine and dental programme students, showed podiatry students suffered the most sharps injuries, with a rate of 31%. Another study showed that only 56% of students reported their injuries, Podiatry practices remain a high‐hazard workplace and under‐reporting of injuries is a widespread problem. Without accurate injury data, effective injury prevention strategies would not be prioritised, leaving podiatrists vulnerable to continued preventable injuries and infection exposures. The first step to putting an effective preventative strategy in place is to increase reporting. This presentation will examine the underlying organisational and individual factors which influence injury reporting rates. We will also explore how evidence‐based changes can be made to empower podiatrists to report their injuries and create sustainable improvements to safety in their organisations. If Molly's incident had been reported, effective prevention strategies would have been introduced to prevent a future occurrence of such injuries. Every contribution to improving the safety culture in podiatry practice, from reporting a personal injury to utilising safety‐engineered devices, helps unleash the potential of change for the benefit of podiatrists, patients, and practices.

## 71 Because You're Worth It: Unleashing Effective Safety Improvements in Podiatry

Michael Sinnott, Chamindika Konara

StaffAndPatientSafety.org

shreya@staffandpatientsafety.org

**ABSTRACT**

‘I dropped the scalpel and landed in my thigh. I continued working as I could see the blood coming through my pants. Once the patient left, I attended to my wound! Too embarrassed to let the patient know what had happened’ (An Australian Podiatrist). Sharps injuries are a serious occupational hazard for podiatrists, students and other staff working in podiatry practices. It is estimated that there are 18,500 sharps injuries per year in Australia. The cuts and injuries can have long‐term impacts on the physical and psychological health of podiatrists, and can be costly for facilities. The healthcare environment is complex and podiatry practice is no different. Podiatrists, in public and private practice, must balance quality of patient care, management pressures, and liaising with external governmental and non‐governmental bodies. In such an environment, there are many competing demands for time and the already scarce resources. As such, podiatrists face significant barriers to create and implement improvements, even when these will improve safety for patients and themselves. In this session, we are going to cover the updates to the regulatory requirements and standards in Australia for sharps handling. We will review the **NHMRC**'s (**National Health Medical Research Council)** Australian Guidelines for the Prevention and Control of Infection in Healthcare updates on sharps safety as required by **APHRA** (**Australian Health Practitioner Regulation Agency**). We will provide practical guidance on prevention strategies following the Hierarchy of Controls. It is vital that all podiatrists who come in contact with sharps are aware of how they can keep themselves and their colleagues safe from injury. In the interactive component of this presentation, we invite knowledge sharing amongst participants to empower podiatrists to implement updates to unleash the potential of safety practices for both, patient and staff safety. This is achieved by collectively designing a practical and effective safety improvement plan. When podiatrists implement these updates to bring about effective safety changes in podiatry practices, we will ensure compliance required by APHRA while unleashing sustainable improvements to both patient and staff safety.

## 72 Lower Limb Kinematics of People With Midfoot Osteoarthritis During Level Walking and Stair Climbing

Merridy J. Lithgow ^1,^*, Jayishni N. Maharaj^2^, Andrew K. Buldt^1^, Shannon E. Munteanu^1^, Benjamin F. Mentiplay^3^, Hylton B. Menz^1^

^1^ *Discipline of Podiatry, School of Allied Health, Human Services and Sport, La Trobe University, Melbourne, Victoria, Australia*, ^2^ *School of Health Sciences and Social Work, Griffith University, Gold Coast, Queensland, Australia*, ^3^ *Discipline of Sport and Exercise Science, School of Allied Health, Human Services and Sport, La Trobe University, Melbourne, Victoria, Australia*

*Merridy.Lithgow@latrobe.edu.au

**Background:** Midfoot osteoarthritis (OA) is a common and disabling condition characterised by arthritic changes in one or more midfoot joints, affecting one in eight people over the age of 50. However, little is known about foot and lower limb kinematics in this population. This study aimed to compare foot and lower limb kinematics between people with and without symptomatic radiographic midfoot OA using a validated lower limb biomechanical model that incorporates tri‐planar foot movements.

**Methods:** Symptomatic radiographic midfoot OA was defined as midfoot pain in the last 4 weeks and radiographic OA in one or more midfoot joints. Cases aged ≥ 45 years were matched 1:1 for sex and age (± 5 years) to controls. A 10‐camera motion analysis system was used to capture foot and lower limb kinematics during level walking and stair climbing, which were analysed with a validated multi‐segmental lower limb model. Group differences were analysed using independent samples *t*‐tests and effect sizes for discrete angles, while statistical parametric mapping compared kinematic patterns between groups.

**Results:** We included 24 midfoot OA cases (mean age 64.4, SD 9.5) matched to 24 controls (mean age 65.2, SD 10.1). During level walking, people with midfoot OA walked slower and displayed absolute joint angles that showed less hip extension throughout stance, less knee flexion in early and late stance, less ankle dorsiflexion throughout stance (medium to large effects), greater subtalar pronation in late stance, and greater tarsometatarsal supination during early stance (medium effects). There were few differences during stair ascent and descent.

**Conclusions:** People with midfoot OA walk slower and demonstrate medium to large differences in sagittal plane hip, knee, and ankle kinematics, and medium differences in subtalar and tarsometatarsal kinematics. These findings provide insights into how people with midfoot OA walk and the mechanisms that may be responsible for OA development.

## 73 Friction Blisters of the Feet: A New Paradigm to Explain Causation

Rebecca Rushton ^1,^*, Douglas Richie^2^

^1^ *Esperance Podiatry & BlisterPod, Esperance, Western Australia, Australia*, ^2^ *California School of Podiatric Medicine, Samuel Merritt University, Oakland, California, USA*

*rebecca@blisterpod.com

Friction blisters of the feet are the most common injury in running, walking, hiking, military training and many sports, and are associated with a 50% increased risk of musculoskeletal injury. Reliable prevention of this skin injury continues to be an elusive quest for both the individual and the treating clinician, underscoring the fact that the pathomechanics of this condition are not fully understood.

The traditional blister causation paradigm revolves around heat, moisture, and friction, with friction denoting a rubbing phenomenon on the skin surface. In reality, foot friction blisters are caused by repetitive shear deformation, with the movement force coming from the bone within the foot, whilst friction force keeps the material interfaces external to the skin surface in stationary contact. The notion of blisters as a superficial‐to‐deep wear injury from external rubbing needs to be abandoned.

Understanding the pathomechanics of the friction blister is essential to effective selection and implementation of prevention strategies, and to understanding the limitations of each prevention strategy. A coherent framework for prevention will be presented to assist practitioners in choosing the most appropriate blister prevention for their clients.

## 74 Factors Associated With Footwear Comfort in Women With Plantar Heel Pain: A Cross‐Sectional Study

Jia Wen Ho^1,2^, Melinda Franettovich Smith^1,3^, Adele van den Hoek^1,4^, Graham Kerr^5^, Sheree Hurn ^1,^*

^1^ *School of Clinical Sciences, Faculty of Health, Queensland University of Technology, Brisbane, Australia*, ^2^ *Department of Podiatry, Sengkang General Hospital, Singapore*, ^3^ *School of Health and Rehabilitation Sciences, Faculty of Health and Behavioural Sciences, The University of Queensland, Brisbane, Australia*, ^4^ *Australian Centre for Health Services Innovation and Centre for Healthcare Transformation, School of Public Health and Social Work, Faculty of Health, Queensland University of Technology, Kelvin Grove, Queensland, Australia*, ^5^ *School of Exercise and Nutrition Sciences, Faculty of Health, Queensland University of Technology, Brisbane, Australia*

*sheree.hurn@qut.edu.au

**Background:** Footwear and insole comfort is crucial for adherence to footwear interventions when treating plantar heel pain (PHP). Higher body mass index (BMI) is a risk factor for PHP, but its relationship with footwear comfort remains unexplored in this population. Although previous studies have indicated that foot posture may be associated with insole preference, this also has not been examined in PHP populations. This study therefore explored factors associated with footwear comfort in women with PHP, including BMI, foot posture and mobility.

**Methods:** BMI, Foot Posture Index (FPI), and foot mobility magnitude were measured in 29 women with PHP (mean age 47 years, range 24–73). Participants walked at a self‐selected speed in six randomised shoe and insole conditions, which varied in terms of outsole and insole material, density and arch contouring. Underfoot comfort was reported (overall, heel, arch and forefoot regions) using a 100 mm visual analogue scale immediately following each condition. For analysis, participants were categorised as having normal (*n* = 17), pronated (*n* = 8), or supinated (*n* = 4) foot posture based on FPI. Participants were secondarily grouped according to their most comfortable shoe condition. Pearson's correlations and one‐way ANOVA were used to investigate associations (*p* < 0.05).

**Results:** Significant inverse correlations were observed between BMI and comfort in certain shoe conditions and regions, indicating that higher BMI was associated with lower footwear comfort. This relationship was observed across all regions in the shoe condition with rubber outsole and flat insole (Pearson's *r* −0.60 to −0.48, *p* < 0.01). Across other shoe conditions, the inverse relationship was most consistent at the forefoot region, with significance observed in five out of six shoe conditions (Pearson's *r* −0.60 to −0.49, *p* < 0.01). One shoe condition which appeared to mitigate the effect included polyurethane outsole and increased arch contouring. Neither FPI nor foot mobility magnitude were associated with comfort scores or most comfortable insole in our sample.

**Conclusions:** This study highlights the importance of BMI as a consideration for footwear comfort in women with PHP. These findings indicate that a cushioned outsole and increased arch contouring may be footwear features to prioritise in women with higher BMI to improve comfort.

## 75 Green Podiatry Student Industry Projects to Prioritise Sustainability and Environmental Responsibility

Steven D. L. Witney^1^, Sarah B. A. R. Fadali^1^, Megan S. K. Chia^1^, Sam I. O. Staifo^1^, Benjamin T. Bourke^1^, Dave W. D. Chang^1^, Angela M. F. Evans ^2,^*

^1^ *School of Allied Health, Human Services and Sport, La Trobe University, Melbourne, Victoria, Australia*, ^2^ *Discipline of Podiatry, School of Allied Health, Human Services and Sport, La Trobe University, Melbourne, Victoria, Australia*

*angela.evans@latrobe.edu.au

**Background:** Climate change harms human health, the environment, and is largely driven by fossil fuels, with the transport sector accounting for 20% of Australia's CO^2^ emissions. Active travel requires good feet (walking, cycling, running), benefits both human and planet health, and should be a primary purpose for podiatry, now and future. This industry project had students explore the connections between feet‐based active travel, health, and carbon emissions.

**Methods:** Following a green podiatry overview, the project commenced. Six students recorded their baseline travel (active or passive) for 2 weeks. The intervention then consisted of maximised active travel for a further 2 weeks. Carbon emissions were examined for each phase.

**Results:** In comparison with baseline active travel, the intervention phase yielded increased active travel for three students (range: 69%–284%, from baseline 100%). In comparison with baseline passive travel, the intervention phase yielded reduced passive travel for five students (range: 51%–114%, from baseline 100%). The 2‐week active travel intervention yielded reduced carbon emissions for five students (range −36,299 to 48,285 g/CO^2^). Overall, there was 36,232 g/CO^2^ emission reduction. Obstacles limiting active travel were identified, viz., access to public transport, personal safety concerns, time constraints.

**Conclusions:** In 2025 an expanded project will include health measures at baseline (BMI, waist girth, BP, HR, sleep, mental health), a 10‐week intervention period, further options, for example increased fruit/vegetables, less red meat and/or ultra‐processed foods.

**Clinical Significance:** Climate change is the greatest threat to health of the 21^st^ century, and is hence immediately relevant to students as future professionals, and citizens. Focus of the benefits of active travel for both human and planet health, enables podiatry to realise the co‐benefits of climate action for public health. Non‐communicable diseases (NCDs) benefit from exercise. A more active and adequately nourished community could reduce NCDs, with advantages for quality of life, and strained healthcare systems. All podiatrists can include physical activity, and basic nutrition, in every initial clinical history interview, utilising applicable standards. To be credible role models, podiatrists need to be active themselves, and visibly participate in community programs, for example Parkrun.

